# Bistability between *π*-diradical open-shell and closed-shell states in indeno[1,2-*a*]fluorene

**DOI:** 10.1038/s41557-023-01431-7

**Published:** 2024-02-08

**Authors:** Shantanu Mishra, Manuel Vilas-Varela, Leonard-Alexander Lieske, Ricardo Ortiz, Shadi Fatayer, Igor Rončević, Florian Albrecht, Thomas Frederiksen, Diego Peña, Leo Gross

**Affiliations:** 1 IBM Research Europe – Zurich, Rüschlikon, Switzerland; 2 Center for Research in Biological Chemistry and Molecular Materials (CiQUS) and Department of Organic Chemistry, University of Santiago de Compostela, Santiago de Compostela, Spain; 3 Donostia International Physics Center (DIPC), Donostia-San Sebastián, Spain; 4 Applied Physics Program, Physical Science and Engineering Division (PSE), King Abdullah University of Science and Technology (KAUST), Thuwal, Kingdom of Saudi Arabia; 5 Department of Chemistry, University of Oxford, Oxford, UK; 6 Ikerbasque, Basque Foundation for Science, Bilbao, Spain

**Keywords:** Scanning probe microscopy, Imaging techniques, Structure elucidation, Electronic properties and materials, Magnetic properties and materials

## Abstract

Indenofluorenes are non-benzenoid conjugated hydrocarbons that have received great interest owing to their unusual electronic structure and potential applications in nonlinear optics and photovoltaics. Here we report the generation of unsubstituted indeno[1,2-*a*]fluorene on various surfaces by the cleavage of two C–H bonds in 7,12-dihydroindeno[1,2-*a*]fluorene through voltage pulses applied by the tip of a combined scanning tunnelling microscope and atomic force microscope. On bilayer NaCl on Au(111), indeno[1,2-*a*]fluorene is in the neutral charge state, but it exhibits charge bistability between neutral and anionic states on the lower-workfunction surfaces of bilayer NaCl on Ag(111) and Cu(111). In the neutral state, indeno[1,2-*a*]fluorene exhibits one of two ground states: an open-shell *π*-diradical state, predicted to be a triplet by density functional and multireference many-body perturbation theory calculations, or a closed-shell state with a *para*-quinodimethane moiety in the *as*-indacene core. We observe switching between open- and closed-shell states of a single molecule by changing its adsorption site on NaCl.

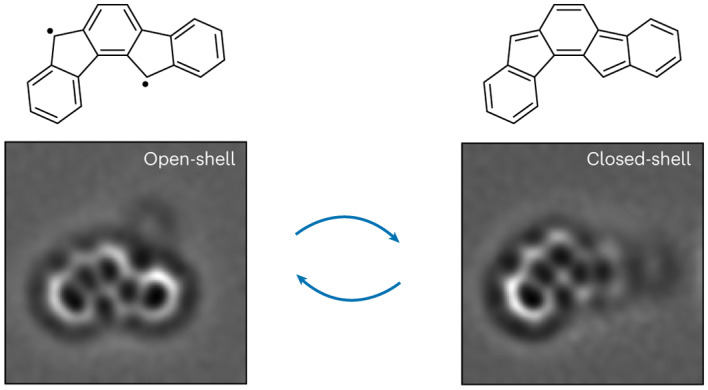

## Abstract (web summary)

Switching the magnetic state of a polycyclic conjugated hydrocarbon in a reversible and controlled manner is challenging. Now, by means of single-molecule scanning probe microscopy, an indenofluorene isomer on ultrathin NaCl films has been shown to adopt both open- and closed-shell states. Furthermore, bidirectional switching between the two states is achieved by changing the adsorption site of the molecule.

## Main

**Fig. 1:**
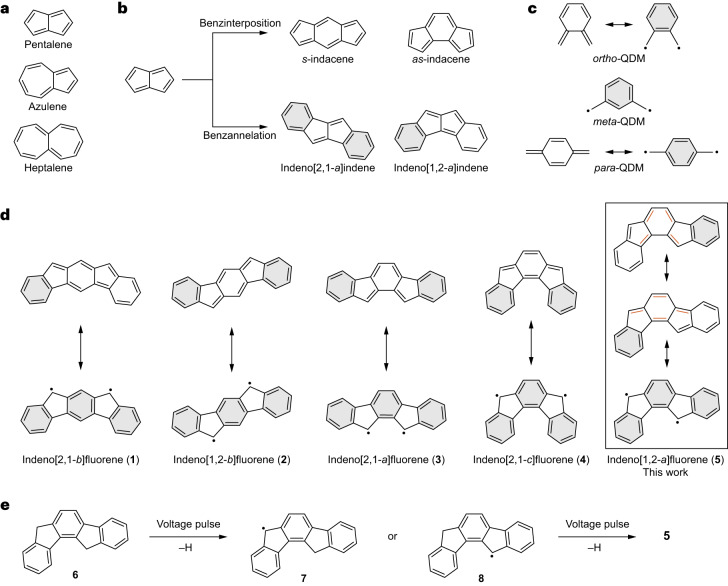
Non-benzenoid non-alternant polycyclic conjugated hydrocarbons. **a**, Classical non-benzenoid non-alternant PCHs: pentalene, azulene and heptalene. **b**, Generation of indacenes and indenoindenes through benzinterposition and benzannelation of pentalene, respectively. Grey filled rings represent Clar sextets. **c**, Closed-shell Kekulé (left) and open-shell non-Kekulé (right) resonance structures of QDMs. Note that *meta*-QDM is a non-Kekulé molecule. All indenofluorene isomers, being derived through benzannelation of indacenes, contain a central QDM moiety. **d**, Closed-shell Kekulé (top) and open-shell non-Kekulé (bottom) resonance structures of indenofluorenes. Compared to their closed-shell structures, **1** and **5** gain two Clar sextets in the open-shell structure, and **2**–**4** gain only one Clar sextet in the open-shell structure. The coloured bonds in **d** highlight the *ortho*- and *para*-QDM moieties in the two closed-shell Kekulé structures of **5**. **e**, Scheme of the on-surface generation of **5** (C_20_H_12_) by voltage pulse-induced dehydrogenation of **6** (C_20_H_14_). Structures **7** and **8** represent the two monoradical species (C_20_H_13_).

The inclusion of non-benzenoid carbocyclic rings is a viable route to tune the physicochemical properties of polycyclic conjugated hydrocarbons (PCHs)^1–3^. Non-benzenoid polycycles may lead to local changes in strain, conjugation and aromaticity, and—relevant to the context of the present work—induce an open-shell ground state of the corresponding PCHs^4–7^. Many non-benzenoid PCHs are also non-alternant, where the presence of odd-membered polycycles breaks the bipartite symmetry of the molecular network^8^. Figure 1a shows classical examples of non-benzenoid non-alternant PCHs, namely, pentalene, azulene and heptalene. Azulene is a stable PCH exhibiting Hückel aromaticity, but pentalene and heptalene are highly reactive Hückel antiaromatic and non-aromatic compounds, respectively. Benzinterposition of pentalene generates indacenes consisting of two isomers, *s*-indacene and *as*-indacene (Fig. 1b). Apart from being antiaromatic, indacenes also contain proaromatic quinodimethane (QDM) moieties (Fig. 1c)^9^, which endow them with potential open-shell character. Although the parent *s*-indacene and *as*-indacene have never been isolated, kinetically and thermodynamically stabilized derivatives of *s*-indacene have been synthesized^10–12^. A feasible strategy to isolate congeners of otherwise unstable non-benzenoid non-alternant PCHs is through fusion of benzenoid rings at the ends of the *π*-system, that is, benzannelation. For example, although the parent pentalene is highly reactive, the benzannelated congener indeno[2,1-*a*]indene is stable under ambient conditions (Fig. 1b)^13^. However, the position of benzannelation is crucial for stability: although indeno[2,1-*a*]indene is stable, its isomer indeno[1,2-*a*]indene (Fig. 1b) oxidizes under ambient conditions^14^. Similarly, benzannelation of indacenes gives rise to the family of PCHs known as indenofluorenes (Fig. 1d), which constitute the topic of the present work. Depending on the benzannelation position and the indacene core, five isomers can be constructed, namely, indeno[2,1-*b*]fluorene (**1**), indeno[1,2-*b*]fluorene (**2**), indeno[2,1-*a*]fluorene (**3**), indeno[2,1-*c*]fluorene (**4**) and indeno[1,2-*a*]fluorene (**5**).

The practical interest in indenofluorenes stems from their low frontier orbital gaps and excellent electrochemical characteristics that render them useful as components in organic electronic devices^15^. The potential open-shell character of indenofluorenes has led to several theoretical studies on their use as nonlinear optical materials^16,17^ and as candidates for singlet fission in organic photovoltaics^18,19^. Recent theoretical work has also shown that indenofluorene-based ladder polymers may exhibit fractionalized excitations^20^. Fundamentally, indenofluorenes represent model systems to study the interplay between aromaticity and magnetism at the molecular scale^17^. Motivated by many of these prospects, the past decade has witnessed intensive synthetic efforts towards the realization of indenofluorenes. Derivatives of **1**–**4** have been realized in solution^21–27^, and **1**–**3**^28–31^ have also been synthesized on surfaces and characterized using scanning tunnelling microscopy (STM) and atomic force microscopy (AFM), which provide information on the molecular orbital densities^32^, molecular structure^33,34^ and oxidation state^35,36^. With regard to the open-shell character of indenofluorenes, **2**–**4** are theoretically and experimentally interpreted to be closed-shell, but calculations indicate that **1** and **5** should exhibit open-shell ground states^17,28,37^. Bulk characterization of mesityl-substituted **1**, including X-ray crystallography, temperature-dependent NMR and electron spin resonance spectroscopy, have provided indications of its open-shell ground state^21^. Electronic characterization of **1** on a Au(111) surface using scanning tunnelling spectroscopy (STS) revealed a low electronic gap of 0.4 eV (ref. ^28^). However, no experimental proof of an open-shell ground state of **1** on Au(111), such as detection of the orbital densities of singly occupied molecular orbitals (SOMOs)^38,39^ or spin excitations and correlations due to unpaired electrons^40,41^, has been shown.

In this Article we report the generation and characterization of unsubstituted **5**. Our research is motivated by theoretical calculations that indicate **5** to exhibit the largest diradical character among all indenofluorene isomers^37^. The same calculations also predict that **5** should possess a triplet ground state. Accordingly, **5** would qualify as a Kekulé triplet, of which only a handful of examples exist^42–44^. However, the synthesis of **5** has remained a challenge. Previously, Dressler and colleagues have reported the transient isolation of mesityl-substituted **5**, but it decomposed both in solution and in the solid state^37^, and only the structural proof of the corresponding dianion was obtained. On-surface generation of a derivative of **5**, starting from truxene as a precursor, was recently reported^45,46^. STM data on this compound, containing the indeno[1,2-*a*]fluorene moiety as part of a larger PCH, were interpreted to indicate its open-shell ground state^46^. However, the results did not imply the ground state of unsubstituted **5**. Here we show that on insulating surfaces (ultrathin NaCl films on (111) coinage metal surfaces), **5** can exhibit one of two ground states: an open shell or a closed shell. We infer the existence of these two ground states based on high-resolution AFM imaging with bond-order discrimination^34^ and STM imaging of molecular orbital densities^32^. AFM imaging reveals molecules with two different geometries. Characteristic bond-order differences in the two geometries concur with the geometry of either an open- or a closed-shell state. Concurrently, STM images at ionic resonances show molecular orbital densities corresponding to SOMOs for the open-shell geometry, but orbital densities of the highest occupied molecular orbital (HOMO) and lowest unoccupied molecular orbital (LUMO) for the closed-shell geometry. Our experimental results are in good agreement with density functional theory (DFT) and multireference perturbation theory calculations. Finally, we observe switching between open- and closed-shell states of a single molecule by changing its adsorption site on the surface.

## Results and discussion

### Synthetic strategy toward indeno[1,2–*a*]fluorene

The generation of **5** relies on the solution-phase synthesis of the precursor 7,12-dihydroindeno[1,2-*a*]fluorene (**6**). Details on the synthesis and characterization of **6** are reported in Supplementary Figs. 1–3. Single molecules of **6** are deposited on coinage metal (Au(111), Ag(111) and Cu(111)) or insulator surfaces. In our work, insulating surfaces correspond to two-monolayers-thick (denoted as bilayer) NaCl on coinage metal surfaces. Voltage pulses ranging between 4 and 6 V are applied by the tip of a combined STM/AFM system, resulting in cleavage of one C–H bond at each of the pentagonal apices of **6**, thereby leading to the generation of **5** (Fig. 1e). In the main text we focus on the generation and characterization of **5** on insulating surfaces. The generation and characterization of **5** on coinage metal surfaces are presented in Supplementary Fig. 4.

### Generation and characterization of indeno[1,2-*a*]fluorene

**Fig. 2:**
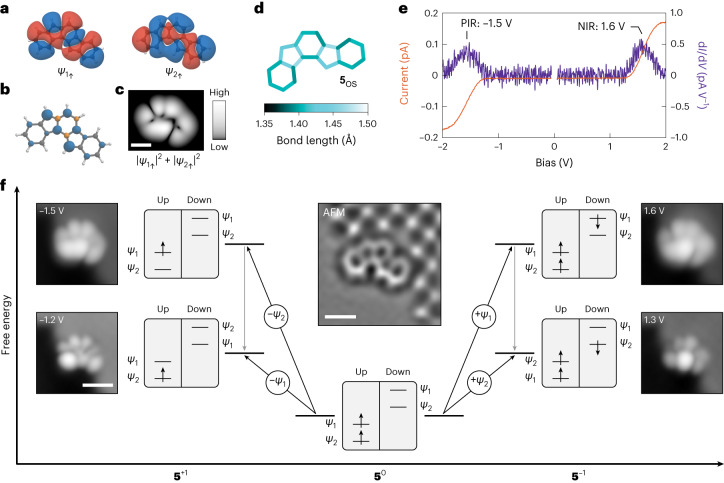
Characterization of open-shell indeno[1,2-*a*]fluorene on bilayer NaCl/Au(111). **a**, DFT-calculated wavefunctions of the frontier orbitals of **5**_OS_ in the triplet configuration for the spin up (occupied) levels (isovalue, 0.002 e^−^ Å^−3^). Blue and red colours represent opposite phases of the wavefunction. Orbital densities (wavefunctions squared) are presented in Supplementary Fig. 12. **b**, Corresponding DFT-calculated spin density of **5**_OS_ (isovalue, 0.01 e^−^ Å^−3^). Blue and orange colours represent spin up and spin down densities, respectively. **c**, Mean-field Hubbard local density of states map of the superposition of the SOMOs of **5**_OS_, calculated at a height of 7 Å above the molecular plane. **d**, DFT-calculated bond lengths of **5**_OS_. **e**, Constant-height *I*(*V*) spectra acquired on a species of **5** assigned as **5**_OS_, along with the corresponding d*I*/d*V*(*V*) spectra. Open feedback parameters: *V* = −2 V, *I* = 0.17 pA (negative bias side) and *V* = 2 V, *I* = 0.17 pA (positive bias side). The acquisition position of the spectra is shown in Supplementary Fig. 7. **f**, Scheme of many-body transitions associated with the measured ionic resonances of **5**_OS_, together with STM images of assigned **5**_OS_ at biases where the corresponding transitions become accessible. Scanning parameters: *I* = 0.3 pA (*V* = −1.2 V and −1.5 V) and 0.2 pA (*V* = 1.3 V and 1.6 V). Centre inset: Laplace-filtered AFM image of assigned **5**_OS_. STM setpoint: *V* = 0.2 V, *I* = 0.5 pA on bilayer NaCl, Δ*z* = −0.3 Å. The tip-height offset, Δ*z*, is provided with respect to the STM setpoint, and positive (negative) values of Δ*z* denote tip approach (retraction) from the STM setpoint. The STM and AFM images shown in **f** are of the same molecule at the same adsorption site, which is next to a third-layer NaCl island. The bright and dark features in the third-layer NaCl island in the AFM image correspond to Cl^−^ and Na^+^ ions, respectively. The STS data shown in **e** were acquired on the molecule shown in **f**. Scale bars, 5 Å (simulated local density of states map and AFM image) and 10 Å (STM images). Source data

**Fig. 3:**
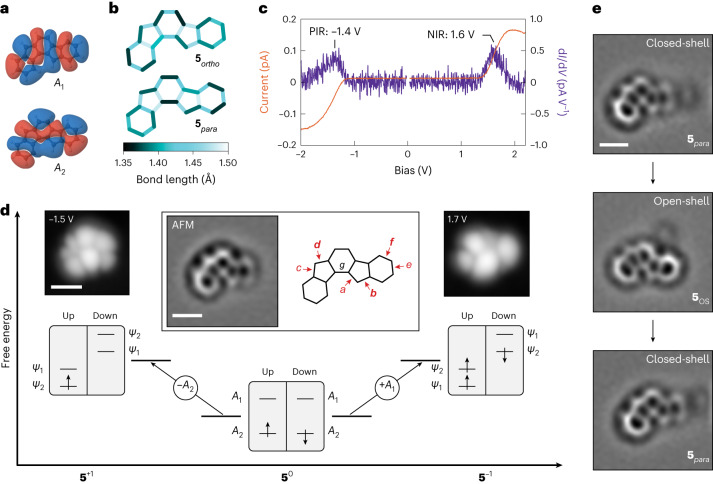
Characterization of closed-shell indeno[1,2-*a*]fluorene on bilayer NaCl/Au(111). **a**, DFT-calculated wavefunctions of the frontier orbitals of closed-shell **5**^0^ (isovalue, 0.002 e^−^ Å^−3^). The wavefunctions shown here are calculated for the **5**_*para*_ geometry. Orbital densities (wavefunctions squared) are presented in Supplementary Fig. 12. **b**, DFT-calculated bond lengths of **5**_*ortho*_ (top) and **5**_*para*_ (bottom). **c**, Constant-height *I*(*V*) spectra acquired on a species of **5** assigned as **5**_*para*_, along with the corresponding d*I*/d*V*(*V*) spectra. Open feedback parameters: *V* = −2 V, *I* = 0.15 pA (negative bias side) and *V* = 2.2 V, *I* = 0.15 pA (positive bias side). The acquisition position of the spectra is shown in Supplementary Fig. 7. **d**, Scheme of many-body transitions associated with the measured ionic resonances of **5**_*para*_, together with STM images of assigned **5**_*para*_ at biases where the corresponding transitions become accessible. Scanning parameters: *I* = 0.15 pA (*V* = −1.5 V) and 0.2 pA (*V* = 1.7 V). Centre inset: Laplace-filtered AFM image of assigned **5**_*para*_. STM setpoint: *V* = 0.2 V, *I* = 0.5 pA on bilayer NaCl, Δ*z* = −0.7 Å. Here, the molecule is adsorbed on top of a defect on the surface. For an example of a **5**_*para*_ species adsorbed adjacent to a third-layer NaCl island, see Supplementary Fig. 15. Also shown in the inset are seven bonds labelled *a*–*g* to highlight the bond-order differences between **5**_*para*_ and **5**_*ortho*_. For the bond pairs *a*/*b*, *c*/*d* and *e*/*f*, the bonds labelled in bold exhibit a higher bond order than their neighbouring labelled bonds in **5**_*para*_. **e**, Laplace-filtered AFM images of **5** on bilayer NaCl/Cu(111) showing switching between **5**_OS_ and **5**_*para*_ as the molecule changes its adsorption position. Switching from **5**_*para*_ to **5**_OS_ was induced by scanning at 1.1 V, while switching from **5**_OS_ back to **5**_*para*_ took place by scanning at −2.2 V. The faint protrusion adjacent to **5** is a defect that stabilizes the adsorption of **5**. STM setpoint: *V* = 0.2 V, *I* = 0.5 pA on bilayer NaCl, Δ*z* = −0.3 Å. STS and STM data in **c** and **d**, respectively, are acquired on the same molecule, whereas the AFM image in **d** is acquired on a different molecule. Scale bars, 5 Å (AFM images) and 10 Å (STM images). Source data

To experimentally explore the electronic structure of **5**, we used bilayer NaCl films on coinage metal surfaces to electronically decouple the molecule from the metal surfaces. Before presenting experimental findings, we summarize the results of our theoretical calculations performed on **5** in the neutral charge state (denoted **5**^0^). We start by performing DFT calculations on **5**^0^ in the gas phase. Geometry optimization performed at the spin-unrestricted UB3LYP/6-31G level of theory leads to one local minimum, **5**_OS_, the geometry of which corresponds to the open-shell resonance structure of **5** (Fig. 1d, Fig. 2d and Supplementary Tables 1–7; the label OS denotes open-shell). The triplet electronic configuration of **5**_OS_ is the lowest-energy state, with the open-shell singlet configuration 90 meV higher in energy. Geometry optimization performed at the restricted closed-shell RB3LYP/6-31G level reveals two local minima, **5**_*para*_ and **5**_*ortho*_, the geometries of which (Fig. 3b) exhibit bond-length alternations in line with the presence of a *para*- or an *ortho*-QDM moiety, respectively, in the *as*-indacene core of the closed-shell resonance structures of **5** (Fig. 1d)^37^. Relative to **5**_OS_ in the triplet configuration, **5**_*para*_ and **5**_*ortho*_ are 0.40 and 0.43 eV higher in energy, respectively. Additional DFT results are shown in Supplementary Fig. 5. To gain more accurate insights into the theoretical electronic structure of **5**, we performed multireference perturbation theory calculations (Supplementary Fig. 6) based on quasi-degenerate second-order *n*-electron valence state perturbation theory (QD-NEVPT2). In so far as the order of the ground and excited states are concerned, the results of QD-NEVPT2 calculations qualitatively match the DFT calculations. For **5**_OS_, the triplet configuration remains the lowest-energy state, with the open-shell singlet configuration 60 meV higher in energy. The energy differences between the open- and closed-shell states are substantially reduced in QD-NEVPT2 calculations, with **5**_*para*_ and **5**_*ortho*_ only 0.11 and 0.21 eV higher in energy, respectively, compared to **5**_OS_ in the triplet configuration. We also performed nucleus-independent chemical shift calculations to probe the local aromaticity of **5** in the open- and closed-shell states. Although **5**_OS_ in the triplet configuration exhibits local aromaticity at the terminal benzenoid rings, **5**_OS_ in the open-shell singlet configuration, **5**_*para*_ and **5**_*ortho*_ all display antiaromaticity (Supplementary Fig. 6).

The choice of the insulating surface determines the charge state of **5**: whereas **5** adopts a neutral charge state on the high-workfunction bilayer NaCl/Au(111) surface (irrespective of its open- or closed-shell state; Supplementary Fig. 7), it exhibits charge bistability between **5**^0^ and the anionic state **5**^−1^ on the lower-workfunction bilayer NaCl/Ag(111) and Cu(111) surfaces (Supplementary Figs. 8 and 9). In the main text, we focus on the characterization of **5** on bilayer NaCl/Au(111). Characterization of charge bistable **5** is reported in Supplementary Figs. 10 and 11. We first describe experiments on **5** on bilayer NaCl/Au(111), where **5** exhibits a geometry corresponding to the calculated **5**_OS_ geometry, and an open-shell electronic configuration. We compare the experimental data on this species to calculations on **5**_OS_ with a triplet configuration, as theory predicts a triplet ground state for **5**_OS_. For **5**_OS_, the calculated frontier orbitals correspond to the SOMOs *ψ*_1_ and *ψ*_2_ (Fig. 2a–c and Supplementary Fig. 12), whose spin up levels are occupied but the spin down levels are empty. Figure 2d shows the DFT-calculated bond lengths of **5**_OS_, where the two salient features, namely the small difference in bond lengths within each ring and the notably longer bond lengths in the pentagonal rings, agree with the open-shell resonance structure of **5** (Fig. 1d).

The inset of Fig. 2f shows an AFM image of **5** adsorbed on bilayer NaCl/Au(111) that we assign as **5**_OS_, where the bond-order differences qualitatively correspond to the calculated **5**_OS_ geometry (discussed and compared to the closed-shell state below). Differential conductance spectra (d*I*/d*V*(*V*), where *I* and *V* denote the tunnelling current and bias voltage, respectively) acquired on assigned **5**_OS_ exhibit two peaks centred at −1.5 V and 1.6 V (Fig. 2e), which we assign to the positive and negative ion resonances (PIR and NIR), respectively. Figure 2f shows the corresponding STM images acquired at the onset (*V* = −1.2 V/1.3 V) and peak (*V* = −1.5 V/1.6 V) of the ionic resonances. To draw a correspondence between the STM images and the molecular orbital densities, we consider tunnelling events as many-body electronic transitions between different charge states of **5**_OS_ (Fig. 2f). Within this framework, the PIR corresponds to transitions between **5**^0^ and the cationic state **5**^+1^. At the onset of the PIR at −1.2 V, an electron can only be detached from the SOMO *ψ*_1_, and the corresponding STM image at −1.2 V shows the orbital density of *ψ*_1_. Increasing the bias to the peak of the PIR at −1.5 V, it becomes possible to also empty the SOMO *ψ*_2_, such that the corresponding STM image shows the superposition of *ψ*_1_ and *ψ*_2_, that is, |*ψ*_1_|^2^ + |*ψ*_2_|^2^ (ref. ^39^). Similarly, the NIR corresponds to transitions between **5**^0^ and **5**^–1^. At the NIR onset of 1.3 V, only electron attachment to *ψ*_2_ is energetically possible. At 1.6 V, electron attachment to *ψ*_1_ also becomes possible, and the corresponding STM image shows the superposition of *ψ*_1_ and *ψ*_2_. The observation of the orbital densities of SOMOs, and not the hybridized HOMO and LUMO, proves the open-shell ground state of assigned **5**_OS_. Measurements of the monoradical species with a doublet ground state are shown in Supplementary Fig. 13.

Unexpectedly, another species of **5** was also experimentally observed that exhibited a closed-shell ground state. In contrast to **5**_OS_, where the frontier orbitals correspond to the SOMOs *ψ*_1_ and *ψ*_2_, DFT calculations predict orbitals of different shapes and symmetries for **5**_*para*_ and **5**_*ortho*_, denoted as *A*_1_ and *A*_2_ and shown in Fig. 3a and Supplementary Fig. 12. For **5**_*ortho*_, *A*_1_ and *A*_2_ correspond to HOMO and LUMO, respectively. The orbitals are inverted in energy and occupation for **5**_*para*_, where *A*_2_ is the HOMO and *A*_1_ is the LUMO. The inset of Fig. 3d shows an AFM image of **5** that we assign as **5**_*para*_. We experimentally infer its closed-shell state first by using qualitative bond-order discrimination by AFM. In high-resolution AFM imaging, chemical bonds with higher bond order are imaged more brightly (that is, with higher frequency shift, Δ*f*) due to stronger repulsive forces, and they appear shorter^34,47,48^. In the inset of Fig. 3d, we also show seven labelled bonds whose bond orders show notable qualitative differences in the calculated **5**_*ortho*_, **5**_*para*_ (Fig. 3b) and **5**_OS_ (Fig. 2d) geometries. In **5**_*para*_, bonds *b* and *d* exhibit a higher bond order than *a* and *c*, respectively. This pattern is reversed for **5**_*ortho*_, whereas the bond orders of bonds *a*–*d* are all similar and small for **5**_OS_. Furthermore, in **5**_*para*_, bond *f* exhibits a higher bond order than *e*, whereas in **5**_*ortho*_ and **5**_OS_, bonds *e* and *f* exhibit similar bond order (because they belong to Clar sextets). Finally, the bond labelled *g* shows a higher bond order in **5**_*para*_ than in **5**_*ortho*_ and **5**_OS_. The AFM image of assigned **5**_*para*_ shown in the inset of Fig. 3d indicates higher bond orders of the bonds *b*, *d* and *f* compared to *a*, *c* and *e*, respectively. In addition, bond *g* appears almost point-like, and with enhanced Δ*f* contrast compared to its neighbouring bonds, indicative of a high bond order (see Supplementary Fig. 14 for height-dependent measurements). These observations concur with the calculated **5**_*para*_ geometry (Fig. 3b). Importantly, all these distinguishing bond-order differences are distinctly different in the AFM image of **5**_OS_ (Fig. 2f, inset), which is consistent with the calculated **5**_OS_ geometry (Fig. 2d). In the AFM images of **5**_OS_ (Fig. 2f, inset and Supplementary Fig. 10), bonds *a*–*d* at the pentagon apices appear with similar contrast and apparent bond length. Bonds *e* and *f*, at one of the terminal benzenoid rings, also exhibit a similar contrast and apparent bond length, and the central bond *g* appears longer compared to assigned **5**_*para*_.

Further compelling evidence for the closed-shell state of assigned **5**_*para*_ is obtained by STM and STS. d*I*/d*V*(*V*) spectra acquired on an assigned **5**_*para*_ species exhibit two peaks centred at −1.4 V (PIR) and 1.6 V (NIR) (Fig. 3c). STM images acquired at these biases (Fig. 3d) show orbital densities of *A*_2_ (PIR) and *A*_1_ (NIR). First, the observation of *A*_1_ and *A*_2_ (and not the SOMOs) as the frontier orbitals of this species strongly indicates its closed-shell state. Second, consistent with the AFM measurements, which indicate good correspondence to the calculated **5**_*para*_ geometry, we observe *A*_2_ as the HOMO and *A*_1_ as the LUMO. For **5**_*ortho*_, *A*_1_ should be observed as the HOMO and *A*_2_ as the LUMO. We did not observe molecules with the signatures of **5**_*ortho*_ in our experiments.

We observed molecules in open-shell (**5**_OS_; Fig. 2) and closed-shell (**5**_*para*_; Fig. 3) states in a similar occurrence after their generation from **6** on the surface (of 47 molecules, 23 and 24 molecules corresponded to **5**_OS_ and **5**_*para*_, respectively). We could also switch individual molecules between the open- and closed-shell states, as shown in Fig. 3e and Supplementary Fig. 15. To this end, a change in the adsorption site of a molecule (whether **5**_OS_ or **5**_*para*_) was induced by STM imaging at either of the ionic resonances, which often resulted in movement of the molecule. The example presented in Fig. 3e shows a molecule that was switched from **5**_*para*_ to **5**_OS_, and back to **5**_*para*_. The switching is not directed; that is, we cannot choose which of the two species will be formed when changing the adsorption site. Out of 22 instances where the molecules moved, 14 resulted in switching between **5**_OS_ and **5**_*para*_, and in eight instances there was no switching of the ground state. Furthermore, we observed **5**_OS_ and **5**_*para*_ in equal yields upon changing the adsorption site. The molecule in the inset of Fig. 3d is adsorbed on top of a defect that stabilizes its adsorption geometry on bilayer NaCl. At defect-free adsorption sites on bilayer NaCl, that is, without a third-layer NaCl island or atomic defects in the vicinity of the molecule, **5** could be stably imaged neither by AFM nor by STM at ionic resonances (Supplementary Fig. 9). Without changing the adsorption site, the state of **5** (open or closed shell) never changed, including in the experiments on bilayer NaCl/Ag(111) and Cu(111), on which the charge state of **5** could be switched (Supplementary Figs. 8 and 9). Also on these lower-workfunction surfaces, both open- and closed-shell species were observed for **5**^0^, and both showed charge bistability^36^ between **5**^0^ (**5**_OS_ or **5**_*para*_) and **5**^−1^ (Supplementary Figs. 10 and 11). The geometrical structure of **5**^−1^ probed by AFM, and its electronic structure probed by STM imaging at the NIR (corresponding to transitions between **5**^−1^ and the dianionic state **5**^−2^), are identical within measurement accuracy for the charged species of both **5**_OS_ and **5**_*para*_. When cycling the charge state of **5** between **5**^0^ and **5**^−1^ several times, we always observed the same state (**5**_OS_ or **5**_*para*_) when returning to **5**^0^, provided the molecule did not move during the charging/discharging process. For a discussion pertaining to the stabilization of and switching between the open- and closed-shell states of **5**, see Supplementary Note 1, and Supplementary Figs. 16 and 17.

## Conclusions

Based on our experimental observations, we conclude that indeno[1,2-*a*]fluorene (**5**) can be stabilized in and switched between an open-shell (**5**_OS_) and a closed-shell (**5**_*para*_) state on NaCl. For the former, both DFT and QD-NEVPT2 calculations predict a triplet electronic configuration. Therefore, **5** can be considered to exhibit the spin-crossover effect, involving magnetic switching between high-spin (**5**_OS_) and low-spin (**5**_*para*_) states, coupled with a reversible structural transformation. So far, the spin-crossover effect has mainly been observed in transition-metal-based coordination compounds with near-octahedral geometry^49^, with relatively few examples of PCHs exhibiting the effect^50^. The observation that the switching between open- and closed-shell states is related to changes in the adsorption site but is not achieved by charge-state cycling alone, indicates that the NaCl surface and local defects facilitate different electronic configurations of **5** depending on the adsorption site. Gas-phase QD-NEVPT2 calculations predict that **5**_OS_ is the ground state, and the closed-shell **5**_*para*_ and **5**_*ortho*_ states are 0.11 and 0.21 eV higher in energy. The experiments, showing bidirectional switching between **5**_OS_ and **5**_*para*_, indicate that a change in the adsorption site can induce sufficient change in the geometry of **5** (leading to a corresponding change in the ground-state electronic configuration) and thus induce switching. Switching between open- and closed-shell states in **5** does not require the formation or dissociation of covalent bonds^51^, but a change of adsorption site on NaCl where the molecule is physisorbed.

Our results should have implications for single-molecule devices, capitalizing on the altered electronic and chemical properties of a system in *π*-diradical open-shell and closed-shell states such as frontier orbital and singlet–triplet gaps, and chemical reactivity. For possible future applications as a single-molecule switch, it might be possible to also switch between open- and closed-shell states by changing the local electric field, such as by using chargeable adsorbates^52^.

## Methods

### Scanning probe microscopy measurements and sample preparation

STM and AFM measurements were performed in a home-built system operating at base pressures below 1 × 10^−10^ mbar and a base temperature of 5 K. Bias voltages are provided with respect to the sample. All STM, AFM and spectroscopy measurements were performed with carbon monoxide (CO)-functionalized tips. AFM measurements were performed in non-contact mode with a qPlus sensor^53^. The sensor was operated in frequency modulation mode^54^ with a constant oscillation amplitude of 0.5 Å. STM measurements were performed in constant-current mode, AFM measurements were performed in constant-height mode with *V* = 0 V, and *I*(*V*) and Δ*f*(*V*) spectra were acquired in constant-height mode. Positive (negative) values of the tip-height offset, Δ*z*, represent tip approach (retraction) from the STM setpoint. All d*I*/d*V*(*V*) spectra were obtained by numerical differentiation of the corresponding *I*(*V*) spectra. STM and AFM images, and spectroscopy curves, were post-processed using Gaussian low-pass filters.

Au(111), Ag(111) and Cu(111) surfaces were cleaned by iterative cycles of sputtering with Ne^+^ ions and annealing up to 800 K. NaCl was thermally evaporated on Au(111), Ag(111) and Cu(111) surfaces held at 323 K, 303 K and 283 K, respectively. This protocol results in the growth of predominantly bilayer (100)-terminated islands, with a minority of third-layer islands. Submonolayer coverage of **6** on the surfaces was obtained by flashing an oxidized silicon wafer containing the precursor molecules in front of the cold sample in the microscope. CO molecules for tip functionalization were dosed from the gas phase on the cold sample.

### Mean-field Hubbard calculations

Tight-binding/mean-field Hubbard calculations were performed by numerically solving the mean-field Hubbard Hamiltonian with nearest-neighbour hopping:

1 $${\widehat{H}}_{\rm{MFH}}={-t\sum _{\langle i,\,j\rangle ,\,\sigma }{c}_{i,\,\sigma }^{\dagger }{c}_{j,\,\sigma }+U\sum _{i,\,\sigma }\langle {n}_{i,\,\sigma }\rangle {n}_{i,\,\overline{\sigma }}-U\sum _{i}\langle {n}_{i,\,\uparrow }\rangle \langle {n}_{i,\,\downarrow }\rangle }$$

where ${c}_{i,\,\sigma }^{\dagger }$ and ${c}_{j,\,\sigma }$ denote the spin selective (${\sigma \in \left\{\uparrow ,\,\downarrow \right\}}$ with ${\bar{\sigma }\in \left\{\downarrow ,\,\uparrow \right\}}$) creation and annihilation operator at neighbouring sites *i* and *j*, *t* = 2.7 eV is the nearest-neighbour hopping parameter, *U* = 3.5 eV is the on-site Coulomb repulsion, and *n*_*i*__, *σ*_ and 〈*n*_*i*__, *σ*_〉 denote the number operator and mean occupation number at site *i*, respectively. Orbital electron densities, *ρ*, of the *n*th eigenstate with energy *E*_*n*_ have been simulated from the corresponding state vector *a*_*n*__,__ *i*__,__ *σ*_ by

2 $${\rho }_{n,\,\sigma }\left(\bf{r}\right)={\left|\sum _{i}{a}_{n,\,i,\,\sigma }{\phi }_{2{p}_{z}}({\bf{r}}-{{\bf{r}}}_{i})\right|}^{2}$$

where ${\phi }_{2{p}_{z}}$ is the Slater 2*p*_z_ orbital for carbon.

### DFT calculations

Gas-phase DFT was employed using the PSI4 program package^55^. All molecules with different charge (neutral and anionic) and electronic (open- and closed-shell) states were independently investigated. The B3LYP exchange-correlation functional with 6-31G basis set was employed for structural relaxation and single-point energy calculations. Convergence criteria were set to 3 × 10^−4^ eV Å^−1^ for the total forces and 10^−6^ eV for the total energies.

For the on-surface DFT calculations shown in Supplementary Fig. 16, we employed the FHI-aims^56^ package. Molecules in the open- and closed-shell states were first independently investigated in the gas phase. The optimized molecular geometries were then optimized on a 9 × 9 bilayer NaCl slab in a cluster-type calculation. Molecular geometries in the gas phase were optimized with the really tight basis defaults. For the on-surface calculations we used light basis for NaCl atoms, and really tight basis defaults for atoms in the molecule. For structural relaxation, we employed the B3LYP exchange-correlation functional using the Vosko–Wilk–Nusair^57^ local-density approximation, as implemented in the FHI-aims package. In addition, we used the van der Waals scheme by Tkatchenko and Scheffler^58^. The convergence criteria for on-surface calculations were set to 10^−3^ eV Å^−1^ for the total forces and 10^−2^ eV for the total energies. For the NaCl slab, we constrained the atoms at the edges of the slab, while the atoms located in the top NaCl layer away from the edges were allowed to relax.

We also studied **5** in the gas phase and adsorbed on bilayer NaCl in a 5 × 5 surface cell using periodic, plane-wave DFT calculations with VASP^59,60^. We employed the optB86b version of the van der Waals density functional^61–64^, a plane-wave energy cutoff of 600 eV, and a 2 × 2 Monkhorst–Pack *k*-point mesh for the surface cell. The NaCl slab was constructed with a bulk lattice constant of 5.64 Å. Structural relaxations of molecule and top NaCl layer (with fixed bottom layer) were performed until residual forces were below 10^–2^ eV Å^–1^. The VASP-calculated adsorption sites and their qualitative differences in energy for the open- and closed-shell states were found to be consistent with the other DFT calculations shown in the main text and the Supplementary Information.

### Multireference calculations

Multireference calculations were performed on the DFT-optimized geometries using the QD-NEVPT2 level of theory^65,66^, with three singlet roots and one triplet root included in the state-averaged calculation. A (10, 10) active space (that is, 10 electrons in 10 orbitals) was used along with the def2-TZVP basis set^67^. Increasing either the active-space size or expanding the basis set resulted in changes of ~50 meV in the relative energies of the singlet and triplet states. These calculations were performed using the ORCA package^68^.

### Nucleus-independent chemical shift calculations

Isotropic nucleus-independent chemical shift values were evaluated at the centre of each ring using the B3LYP exchange-correlation functional with def2-TZVP basis set using the Gaussian 16 software package^69^.

## Online content

Any methods, additional references, Nature Portfolio reporting summaries, source data, extended data, supplementary information, acknowledgements, peer review information; details of author contributions and competing interests; and statements of data and code availability are available at 10.1038/s41557-023-01431-7.

### Supplementary information

Supplementary Information Supplementary Figs. 1–17, Tables 1–7, Note 1 and Discussion.

Supplementary Data Source data for Supplementary Figs. 5–8, 10 and 11.

### Source data

Source Data Fig. 2 Source data for scanning tunnelling spectroscopy measurements shown in Fig. 2e.

Source Data Fig. 3 Source data for scanning tunnelling spectroscopy measurements shown in Fig. 3c.

## Data Availability

The data that support the findings of this study are available in the paper and its Supplementary Information, which contains materials and methods, solution synthesis and characterization of **6**, additional STM and AFM data of **5**, STM and AFM data of monoradical species, analysis of Δ*f*(*V*) spectra, and additional calculations. Output files of DFT and multireference calculations are available at 10.5281/zenodo.8234159. Source data are provided with this paper.
