# Supplementary material for: Bistability between π-diradical open-shell and closed-shell states in indeno[1,2-a]fluorene
Source: Nat Chem. 2024 Feb 8;16(5):755–61. doi: 10.1038/s41557-023-01431-7 (PMC11087267; doi:10.1038/s41557-023-01431-7)
Supplement: Supplementary file 1 — Supplementary Figs. 1–17, Tables 1–7, Note 1 and Discussion. [file 41557_2023_1431_MOESM1_ESM.pdf]

nature chemistry

Article

https://doi.org/10.1038/s41557-023-01431-7

Bistability between π-diradical open-shell and closed-shell states in indeno[1,2-*a*] fluorene

In the format provided by the authors and unedited

Supplementary Information

Bistability between π-diradical open-shell and closed-shell states in indeno[1,2-*a*]fluorene

Shantanu Mishra[1], Manuel Vilas-Varela[2], Leonard-Alexander Lieske[1], Ricardo Ortiz[3], Shadi Fatayer[4], Igor Rončević[5], Florian Albrecht[1], Thomas Frederiksen[3,6], Diego Peña[2] and Leo Gross[1]

[1]IBM Research Europe – Zurich, 8803 Rüschlikon, Switzerland

[2]Center for Research in Biological Chemistry and Molecular Materials (CiQUS) and Department of Organic Chemistry, University of Santiago de Compostela, 15782 Santiago de Compostela, Spain

[3]Donostia International Physics Center (DIPC), 20018 Donostia-San Sebastián, Spain

[4]Physical Science and Engineering Division, King Abdullah University of Science and Technology (KAUST), 23955-6900 Thuwal, Saudi Arabia

[5]Department of Chemistry, Oxford University, Oxford OX1 3TA, United Kingdom

[6]Ikerbasque, Basque Foundation for Science, 48013 Bilbao, Spain

Contents:

1. Solution synthesis and characterization data. Page 2

2. Scanning probe microscopy and spectroscopy data, and theoretical calculations. Page 5

3. References Page 24

1. Solution synthesis and characterization data.

Experimental details

Starting materials (reagent grade) were purchased from TCI and Sigma-Aldrich and used without further purification. Reactions were carried out in flame-dried glassware and under an inert atmosphere of purified Ar using Schlenk techniques. Thin-layer chromatography was performed on Silica Gel 60 F-254 plates (Merck). Column chromatography was performed on silica gel (40-60 µm). Nuclear magnetic resonance (NMR) spectra were recorded with Bruker Varian Mercury 300 or Bruker Varian Inova 500 spectrometers. Mass spectrometry (MS) data were recorded with a Bruker Micro-TOF spectrometer. The synthesis of compound **6** was developed following the two-step synthetic route shown in Supplementary Fig. 1, which is based on the preparation of methylene-bridge polyarenes by means of Pd-catalyzed activation of benzylic C-H bonds[1].

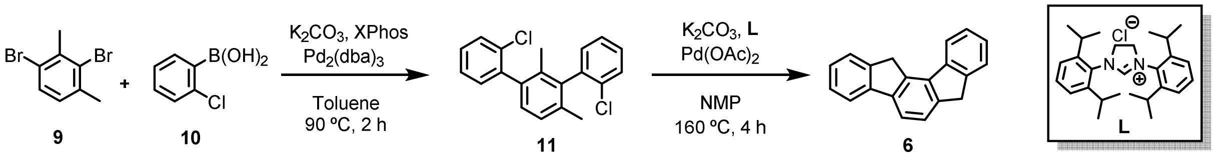

Supplementary Fig. 1 | Synthetic route to obtain compound **6**.

Synthesis of 2,2''-dichloro-2',4'-dimethyl-1,1':3',1''-terphenyl (11)

The complex $Pd_2(dba)_3$ (20 mg, 0.02 mmol) was added over a deoxygenated mixture of 1,3-dibromo-2,4-dimethylbenzene (**9**, 100 mg, 0.38 mmol), (2-chlorophenyl)boronic acid **10** (178 mg, 1.14 mmol), K_2CO_3 (314 mg, 2.28 mmol) and XPhos (35 mg, 0.08 mmol) in toluene (1:1, 10 mL), and the resulting mixture was heated at 90 °C for 2 h. After cooling to room temperature, the solvents were evaporated under reduced pressure. The reaction crude was purified by column chromatography (SiO_2; hexane:CH_2Cl_2 9:1) affording **11** (94 mg, 76%) as a colorless oil. **^{1}H NMR** (300 MHz, 298 K, $CDCl_3$) δ: 7.51 (m, 2H), 7.40 – 7.28 (m, 5H), 7.27 – 7.20 (m, 2H), 7.13 (d, J = 7.7 Hz, 1H), 2.07 (s, 3H), 1.77 (s, 3H) ppm. **^{13}C NMR-DEPT** (75 MHz, 298 K, $CDCl_3$, 1:1 mixture of atropisomers) δ: 141.2 (C), 141.1 (C), 140.0 (C), 139.4 (2C), 137.5 (C), 137.4 (C), 136.0 (3C), 134.8 (C), 134.5 (C), 134.1 (C), 134.0 (C), 133.7 (C), 133.6 (C), 131.6 (CH), 131.2 (CH), 131.1 (CH), 130.7 (CH), 129.8 (CH), 129.7 (CH), 129.5 (CH), 129.4 (CH), 129.0 (CH), 128.9 (CH), 128.7 (2CH), 128.6 (2CH), 127.2 (CH), 127.1 (CH), 127.0 (CH), 126.9 (CH), 126.7 (CH), 126.6 (CH), 20.6 (CH_3), 20.5 (CH_3), 17.7 (CH_3), 17.5 (CH_3) ppm. **MS (APCI)** *m/z* (%): 327 (M+1, 100). **HRMS**: $C_{20}H_{16}Cl_2$; calculated: 327.0702, found: 327.0709.

Synthesis of 7,12-dihydroindeno[1,2–*a*]fluorene (6)

The complex $Pd(OAc)_2$ (7 mg, 0.03 mmol) was added over a deoxygenated mixture of terphenyl **11** (90 mg, 0.27 mmol), K_2CO_3 (114 mg, 0.83 mmol) and ligand **L** (26 mg, 0.06 mmol) in NMP (2 mL). The resulting mixture was heated at 160 °C for 4 h. After cooling to room temperature, H_2O (30 mL) was added, and the mixture was extracted with EtOAc (3x15 mL). The combined organic extracts were dried over anhydrous Na_2SO_4, filtered, and evaporated under reduced pressure. The reaction crude was purified by column chromatography (SiO_2; hexane:CH_2Cl_2 9:1) affording compound **6** (8 mg, 11%) as a white solid. **^{1}H NMR** (500 MHz, 298 K, $CDCl_3$) δ: 7.93 (d, J = 7.6 Hz, 1H), 7.85 (d, J = 7.5 Hz, 1H), 7.78 (d, J = 7.7 Hz, 1H), 7.65 (d, J = 7.4 Hz, 1H), 7.61 (d, J = 7.5 Hz, 1H), 7.59 (d, J = 7.7 Hz, 1H), 7.47 (ddd, J = 8.4, 7.2, 1.1 Hz, 1H), 7.42 (dd, J = 8.1, 7.0 Hz, 1H), 7.35 (m, 2H), 4.22 (s, 3H), 4.02 (s, 3H). ppm. **^{13}C NMR-DEPT** (125 MHz, 298 K, $CDCl_3$) δ: 144.1 (C), 143.3 (C), 142.3 (C), 141.9 (C), 141.8 (C), 141.2 (C), 138.2 (C), 136.5 (C), 127.0 (CH), 126.9 (CH), 126.7 (CH), 126.6 (CH), 125.3 (CH), 125.2 (CH), 123.6 (CH), 122.2 (CH), 119.9 (CH), 118.4 (CH), 37.4 (CH_2), 36.3 (CH_2). ppm. **MS (APCI)** *m/z* (%): 254 (M+, 88). **HRMS**: $C_{20}H_{14}$; calculated: 254.1090, found: 254.1090.

^{1}H and ^{13}C NMR spectra

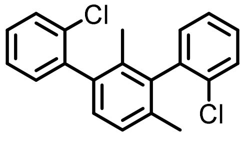

11

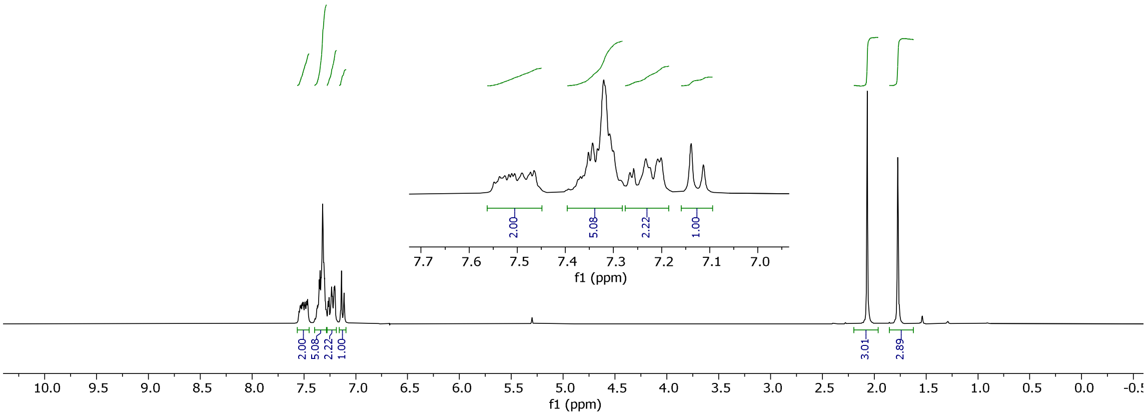

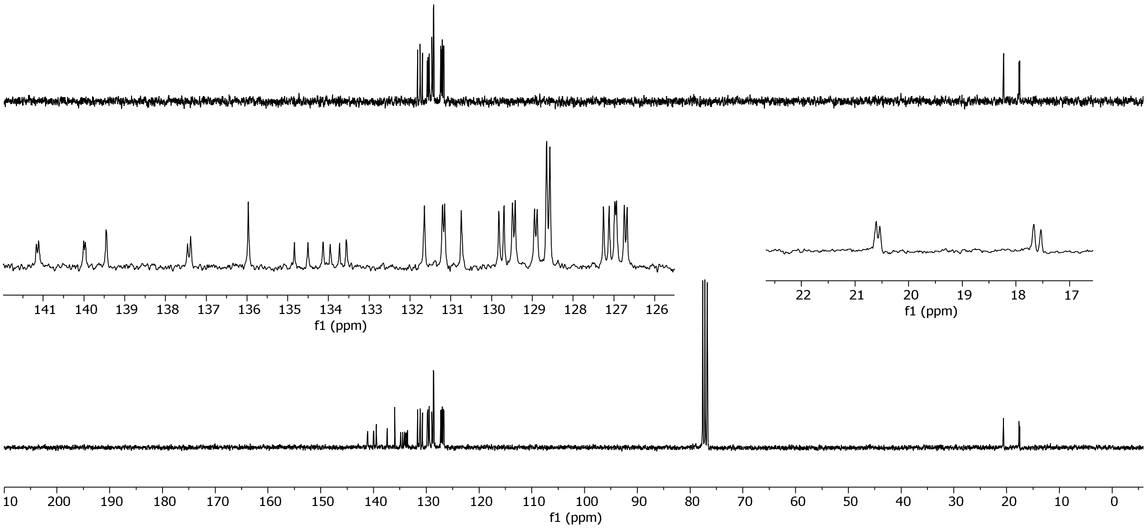

Supplementary Fig. 2 | ^{1}H (300 MHz, 298 K, $CDCl_3$, top) and ^{13}C NMR (75 MHz, 298 K, $CDCl_3$, bottom) spectra of compound **11**.

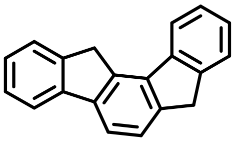

6

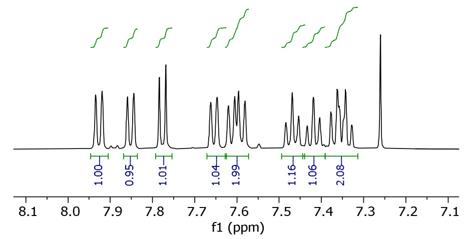

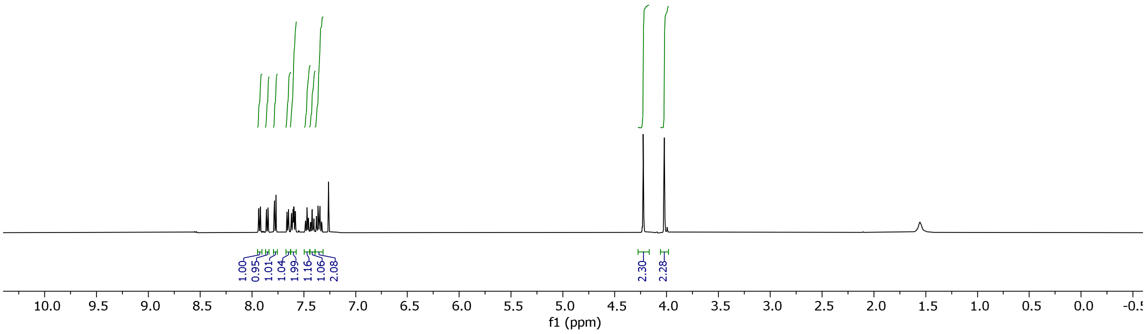

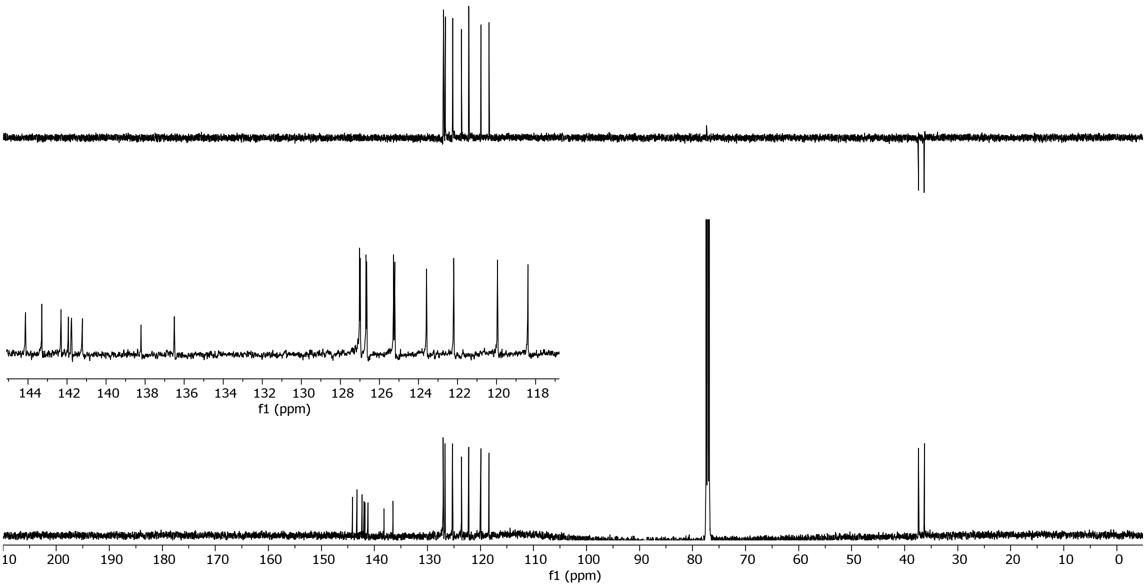

Supplementary Fig. 3 | ^{1}H (500 MHz, 298 K, $CDCl_3$, top) and ^{13}C (125 MHz, 298 K, $CDCl_3$, bottom) NMR spectra of compound **6**.

2. Scanning probe microscopy and spectroscopy data, and theoretical calculations.

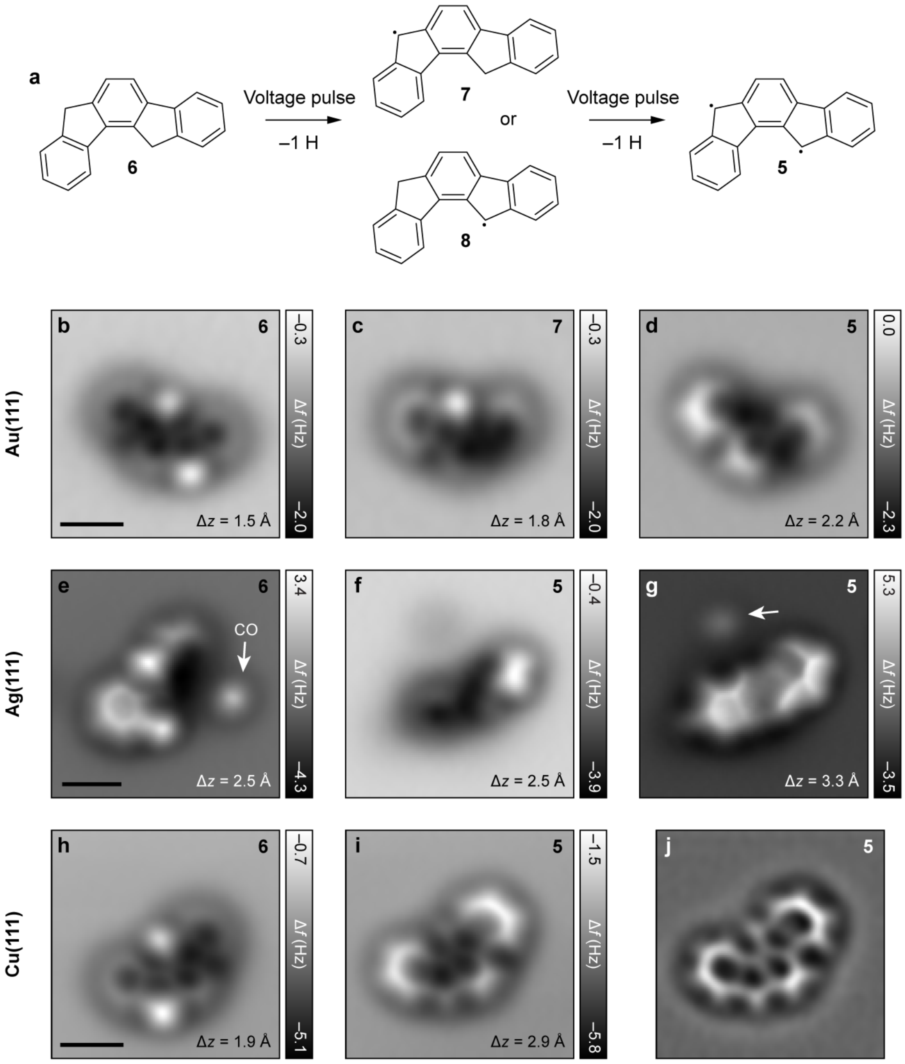

Supplementary Fig. 4 | Generation of indeno[1,2-*a*]fluorene on coinage metal surfaces. a, Scheme of on-surface generation of **5** ($C_{20}H_{12}$) by voltage pulse-induced dehydrogenation of **6** ($C_{20}H_{14}$). Structures **7** and **8** represent the two monoradical species ($C_{20}H_{13}$). To generate **5**, the STM tip was positioned at the center of **6** at typical tunneling conditions of $V = 0.2$ V and $I = 1$ pA, and the feedback loop was switched off to maintain a constant tip height relative to the molecule. The tip was then retracted by 5–10 Å to limit the tunneling current, and the bias was ramped to 4–6 V. Abrupt changes in the $I(V)$ spectra indicated manipulation events, and the area was subsequently imaged to monitor the occurrence of dehydrogenation. **b–d,** AFM images showing stepwise generation of **5** on Au(111). AFM images are shown for precursor **6** (**b**), in which the two bright protrusions correspond to -CH_2- moieties at the pentagonal ring apexes, **7** (**c**), and **5** (**d**). **e–g,** AFM images showing generation of **5** on Ag(111). The arrow in **e** indicates a CO molecule adjacent to **6**, while the arrow in **g** indicates an adsorbate adjacent to **5**. **f** and **g** show AFM images of **5** at two different heights. The tip is 0.8 Å closer to **5** in **g** than in **f**. **h,i,** AFM images showing generation of **5** on Cu(111). **j**, Laplace-filtered version of **i** revealing the carbon skeleton of **5**. On Cu(111), **5** adsorbs in a largely planar geometry, although the increased frequency shift in AFM imaging at the terminal benzenoid rings (leading to their brighter contrast) compared to the central rings implies a slight out-of-plane distortion of **5**. This feature was also observed for pentacene[2] and **2** (ref. [3]) on Cu(111). Additionally, qualitatively similar intramolecular resolution of **5** on Cu(111) is obtained at a much smaller tip-sample distance than on **6** (compare Δz values in **h** and **i**), indicating a substantially reduced adsorption height of **5**. On Au(111) (**d**) and Ag(111) (**g**), **5** adsorbs

in a non-planar conformation, where the apical carbon atoms of the pentagonal rings are not resolved in AFM imaging due to their reduced adsorption height compared to the rest of the carbon atoms. We attribute this observation to the significantly different lattice parameter of Cu(111) (2.57 Å) compared to Au(111) and Ag(111) (2.95 Å and 2.94 Å, respectively)[4], such that the apical carbon atoms of the pentagonal rings of **5** adsorb on the on-top atomic sites on Au(111) and Ag(111), but not on Cu(111). Our speculation is based on a previous study of polymers of **1** on Au(111) by Di Giovannantonio et al.[5], where both tilted and planar individual units of **1** were observed depending on whether the apical carbon atoms of the pentagonal rings in **1** adsorbed on the on-top or hollow sites of the surface, respectively. Given the strong molecule-metal interaction, we found no electronic state signatures of **5** on all three metal surfaces. STM set point for AFM images: V = 0.2 V and I = 0.5 pA on Au(111) and Ag(111), and V = 0.2 V and I = 1 pA on Cu(111). Scale bars: 5 Å.

Supplementary Table 1 | DFT-calculated C–C bond lengths (in Å) of $\mathbf{5}_{\mathrm{OS}}$, $\mathbf{5}_{\mathit{para}}$, $\mathbf{5}_{\mathit{ortho}}$ and $\mathbf{5}^{-1}$. Numbering scheme of carbon atoms is shown in the molecular model.

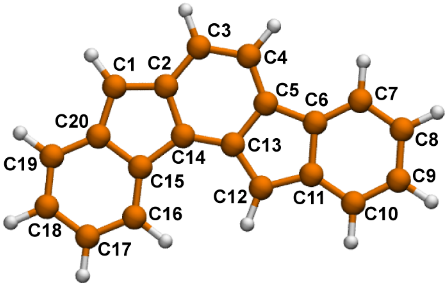

Bond	Bond length, $\mathbf{5}_{\mathrm{OS}}$	Bond length, $\mathbf{5}_{\mathit{para}}$	Bond length, $\mathbf{5}_{\mathit{ortho}}$	Bond length, $\mathbf{5}^{-1}$
C1–C2	1.423	1.380	1.459	1.434
C2–C3	1.412	1.438	1.376	1.417
C3–C4	1.404	1.372	1.439	1.402
C4–C5	1.394	1.429	1.368	1.398
C5–C6	1.465	1.398	1.477	1.450
C6–C7	1.392	1.426	1.385	1.398
C7–C8	1.406	1.374	1.414	1.403
C8–C9	1.405	1.446	1.396	1.410
C9–C10	1.400	1.370	1.412	1.400
C10–C11	1.403	1.437	1.392	1.410
C11–C12	1.439	1.382	1.464	1.437
C12–C13	1.428	1.461	1.387	1.404
C13–C14	1.401	1.368	1.429	1.422
C14–C15	1.466	1.481	1.407	1.444
C15–C16	1.393	1.386	1.422	1.405
C16–C17	1.407	1.415	1.378	1.399
C17–C18	1.404	1.394	1.441	1.415
C18–C19	1.401	1.412	1.371	1.395
C19–C20	1.402	1.390	1.434	1.411
C1–C20	1.441	1.466	1.384	1.423
C2–C14	1.450	1.479	1.461	1.439
C5–C13	1.456	1.476	1.480	1.480
C6–C11	1.439	1.486	1.429	1.450
C15–C20	1.441	1.430	1.483	1.460

Supplementary Table 2 | DFT-optimized gas phase geometry of 5_{OS}.

Atom	X (Å)	Y (Å)	Z (Å)
C	-4.493874905187	1.674619220405	-1.609303916742
C	-3.863487509338	0.528779745012	-2.119253744366
C	-2.611822561254	0.113827348963	-1.629441264251
C	-1.996102060038	0.856210218342	-0.624059854977
C	-2.642110148231	2.032366121432	-0.099920908974
C	-3.889266818531	2.433231576145	-0.598135899742
C	-0.725251505360	0.704435280938	0.090931506587
C	-0.629327165027	1.798198500825	1.037529797146
C	-1.796805091569	2.602862599527	0.917952130093
C	0.318117383988	-0.228299667792	0.025262767673
C	1.461241767848	-0.063930174510	0.911432158857
C	1.536593440951	0.994329799145	1.815800584807
C	0.492527175395	1.930779898123	1.883999318271
C	0.535544659889	-1.393262988491	-0.772313363113
C	1.801567647606	-1.970428907234	-0.404050534403
C	2.387392905819	-1.164000839683	0.633372852920
C	2.475643712545	-3.106613430179	-0.877416915878
C	3.719207610492	-3.437170915929	-0.324772042620
C	4.288121828211	-2.646978444783	0.688394153431
C	3.624504160418	-1.505646622364	1.172808493764
H	-5.459573539461	1.975093163789	-2.003101175612
H	-4.347732620325	-0.045753454284	-2.902426535897
H	-2.144054134383	-0.774996617744	-2.038999819066
H	-4.381361793425	3.318822570739	-0.207227631455
H	-2.015611303951	3.493449520313	1.493020528289
H	2.397744598582	1.099699275791	2.468472126088
H	0.547513427309	2.756240089197	2.586921973707
H	-0.129763282138	-1.786484482106	-1.528387260264
H	2.041280122519	-3.721272006993	-1.660127876839
H	4.250916952706	-4.313266511090	-0.681905848943
H	5.252996532539	-2.921178864985	1.102875782251
H	4.077963948155	-0.905768559282	1.956172095786

Supplementary Table 3 | DFT-optimized gas phase geometry of 5*ortho*.

Atom	X (Å)	Y (Å)	Z (Å)
C	-4.134416460352	2.814309431291	-0.845551534497
C	-3.640407266867	1.809607147554	-1.752460841137
C	-2.510360497149	1.072189732277	-1.473391689028
C	-1.806851583881	1.303250559133	-0.258975697678
C	-2.312412915420	2.336692786536	0.677048697414
C	-3.493359800352	3.076034253535	0.338216034376
C	-0.649964490800	0.731255759410	0.302591894163
C	-0.428804510017	1.399481290028	1.582451710616
C	-1.480453252946	2.390301188423	1.782001753306
C	0.269037218441	-0.286494228615	-0.100827307874
C	1.395180754790	-0.604568725691	0.805767507828
C	1.566313962264	0.045720529979	1.997000128817
C	0.633773775906	1.068723114999	2.391684629095
C	0.373841369116	-1.109111813579	-1.212254070458
C	1.545718854217	-1.975687378697	-1.076443038658
C	2.185713159133	-1.674768742806	0.165113205293
C	2.069992548441	-2.957983016218	-1.912297148076
C	3.236621389901	-3.643427299416	-1.509831233519
C	3.859342671754	-3.347201104010	-0.296602843074
C	3.330548845840	-2.350260448835	0.555771330262
H	-5.029409002597	3.365264701563	-1.116443306242
H	-4.180866951831	1.636844384159	-2.678246526949
H	-2.162794902644	0.322354134391	-2.176237653349
H	-3.867777378661	3.833860524632	1.020069392113
H	-1.576416780992	3.038965035250	2.642011015389
H	2.395531896427	-0.195100598260	2.654599156591
H	0.782863264368	1.576348743889	3.340669582436
H	-0.295003555014	-1.126747683052	-2.062164576745
H	1.595429620084	-3.198218041960	-2.858713191362
H	3.652876510392	-4.410917009506	-2.154747534109
H	4.755412092791	-3.883428054063	-0.002166263143
H	3.822353475338	-2.126845898377	1.498047369205

Supplementary Table 4 | DFT-optimized gas phase geometry of 5*para*.

Atom	X (Å)	Y (Å)	Z (Å)
C	-3.389276477755	3.585428848613	1.027178952043
C	-2.017911766127	3.761323537237	1.208339511802
C	-1.109850316602	2.720208485429	0.903602820347
C	-1.604865259809	1.518734727532	0.420707071205
C	-3.011184710328	1.339298501435	0.235274102316
C	-3.900655192096	2.364535838239	0.535767492019
C	-0.954510753441	0.252430661494	0.011436525006
C	-2.029089753515	-0.668326057043	-0.417758966204
C	-3.236128110607	-0.014627973278	-0.280584333231
C	0.346664182123	-0.166335624388	-0.031541925290
C	0.638055604807	-1.532834663686	-0.508100210036
C	-0.413615402248	-2.408615945404	-0.918844831197
C	-1.721665159283	-1.995607808125	-0.878565605522
C	1.611807843022	0.478726096524	0.312714580776
C	2.620301782542	-0.433034686700	0.063970396618
C	2.025852534361	-1.694234541302	-0.449290043167
C	4.046019322961	-0.346354975939	0.217019143185
C	4.822235481152	-1.425350465747	-0.112831769245
C	4.239499962828	-2.650729855385	-0.611951360678
C	2.882498483024	-2.786382815714	-0.777121632266
H	-4.072975836230	4.393796135899	1.265607288704
H	-1.638949024555	4.704909994818	1.586752830020
H	-0.046992027060	2.875089158012	1.050818837036
H	-4.969695701787	2.235705394866	0.397584642917
H	-4.210599358659	-0.425823864268	-0.514319373744
H	-0.168315192456	-3.408409956239	-1.264787561340
H	-2.527393729101	-2.652458007972	-1.188074530586
H	1.732136836821	1.482774972453	0.691313236552
H	4.493112322774	0.569677068469	0.591114355720
H	5.900946359438	-1.374046695575	-0.002276088703
H	4.901228762556	-3.475252948843	-0.858178735430
H	2.462791307205	-3.714456719140	-1.153306535775

Supplementary Table 5 | DFT-optimized gas phase geometry of 5^{-1}.

Atom	X (Å)	Y (Å)	Z (Å)
C	-4.496738578962	1.708824260175	-1.625653354891
C	-3.860788175699	0.552835298733	-2.136595005000
C	-2.619380304249	0.131698446179	-1.646823114410
C	-1.986038554152	0.864630303977	-0.628974113388
C	-2.641794262136	2.057311186331	-0.101552006701
C	-3.893117894423	2.457441931798	-0.615010167601
C	-0.735996557671	0.699601012078	0.074202202667
C	-0.637138866908	1.781767884751	1.016869378518
C	-1.803496285863	2.608932409589	0.906708726568
C	0.308082418908	-0.262273057992	-0.006163081082
C	1.458897501728	-0.084477836306	0.907924472745
C	1.522072131459	0.980185861855	1.812501362788
C	0.481585604013	1.917253649910	1.875215511143
C	0.536566551744	-1.409580941133	-0.782787607119
C	1.798088142965	-1.981235777145	-0.401323854729
C	2.384326459236	-1.170026293088	0.648087547418
C	2.503603915808	-3.116854258233	-0.848918209301
C	3.741497819863	-3.436105292764	-0.278935700341
C	4.306091133464	-2.643244699273	0.741125191822
C	3.621844781317	-1.508181538623	1.202477941900
H	-5.461516556915	2.012257346928	-2.025584355764
H	-4.347321051188	-0.017937842520	-2.924724031879
H	-2.150661305504	-0.758834906002	-2.054583141165
H	-4.384184971344	3.346668950402	-0.223180959028
H	-2.007108641425	3.498870929015	1.491568984299
H	2.382142148672	1.083088794982	2.471482458596
H	0.531696235301	2.745005116626	2.579317754140
H	-0.117227905498	-1.810899782963	-1.545558825564
H	2.088913476297	-3.744662625798	-1.635019178226
H	4.279923557751	-4.314780664127	-0.630261857185
H	5.269103724923	-2.911691323034	1.168251145415
H	4.057135025593	-0.895278424907	1.990248785612

Supplementary Table 6 | DFT-optimized gas phase geometry of 7.

Atom	X (Å)	Y (Å)	Z (Å)
C	-4.491439050731	1.686733076122	-1.602351083526
C	-3.841797181294	0.563790955315	-2.139387665174
C	-2.590597686532	0.147470862289	-1.646338756751
C	-1.997928821386	0.865769800635	-0.612182179376
C	-2.661250902060	2.018049494648	-0.058884345566
C	-3.908595381434	2.420326640321	-0.561493874636
C	-0.730243124316	0.703438641421	0.114531537399
C	-0.656750745004	1.766301987286	1.094523200853
C	-1.835580426518	2.564732651717	0.984341833637
C	0.306786430404	-0.206852900546	0.029205368409
C	1.426912711730	-0.081650146050	0.906497106243
C	1.498394164817	0.949651289874	1.857524840894
C	0.456246192539	1.875187605716	1.952733192436
C	0.498236847232	-1.403705046747	-0.886169970705
C	1.844875798271	-1.953532887941	-0.444208826468
C	2.370594585872	-1.161901906850	0.608408033756
C	2.551572900101	-3.053406542944	-0.921827487179
C	3.793194338892	-3.368458565480	-0.346476707894
C	4.315515234352	-2.586632773114	0.695247599346
C	3.609677269274	-1.479576712044	1.179740779816
H	-5.455584141790	1.988442785556	-1.999050627797
H	-4.309910509456	0.008278802428	-2.945853318944
H	-2.105520571071	-0.723305284345	-2.075436944232
H	-4.415655203512	3.288065981699	-0.150142953476
H	-2.070356879362	3.434812229294	1.584175582337
H	2.357407247711	1.029508512452	2.516068778035
H	0.503084361149	2.675689992473	2.684819462637
H	0.501096408583	-1.112315434107	-1.946251605008
H	-0.306664902104	-2.143118963990	-0.767463772690
H	2.153787888007	-3.663303138252	-1.727823227019
H	4.353531271394	-4.223780526291	-0.710512318882
H	5.276701747150	-2.843541320174	1.129279598838
H	4.020275760465	-0.879500991232	1.985954283129

Supplementary Table 7 | DFT-optimized gas phase geometry of 8.

Atom	X (Å)	Y (Å)	Z (Å)
C	-4.463433722451	1.611654029028	-1.664386043649
C	-3.790702034001	0.486640116448	-2.163076605258
C	-2.548521077631	0.105981347855	-1.642201879526
C	-1.979676097998	0.865482796512	-0.610178282647
C	-2.664872584464	2.003277027704	-0.109100857066
C	-3.899765579209	2.376533026953	-0.631310857412
C	-0.716577433729	0.719352085761	0.124191937844
C	-0.623954968623	1.768633447154	1.078758286441
C	-1.854288357225	2.655330911749	0.995937533527
C	0.331043365969	-0.219734961122	0.038302428371
C	1.462689063781	-0.078862492708	0.927202526738
C	1.529470173495	0.954987499332	1.851091732529
C	0.473589365972	1.890110064877	1.927998522489
C	0.549422786206	-1.368142464194	-0.789093079013
C	1.811558595909	-1.955941128370	-0.430687445108
C	2.393805587106	-1.176432951489	0.627448373949
C	2.486553598499	-3.082236072209	-0.928941376375
C	3.727163147498	-3.428142215763	-0.379614603545
C	4.292009724505	-2.663143515602	0.654366291687
C	3.626543671300	-1.531924641899	1.163280033419
H	-5.425959981307	1.892449952142	-2.080031003780
H	-4.238056922627	-0.096026126918	-2.962261416191
H	-2.042848891733	-0.766367590174	-2.039751658099
H	-4.424138071671	3.247137611742	-0.247956749064
H	-1.595703706496	3.696144914104	0.754591509281
H	-2.405815560554	2.683399891509	1.946430895343
H	2.383560640370	1.052420109123	2.514419443040
H	0.523090363047	2.699911152815	2.649759768305
H	-0.111933778860	-1.743341796303	-1.557439983697
H	2.054510817892	-3.677185620334	-1.728154116732
H	4.259182841771	-4.296185145194	-0.755602373511
H	5.254777891001	-2.947674347760	1.066994632341
H	4.077883319184	-0.951949093358	1.962887011741

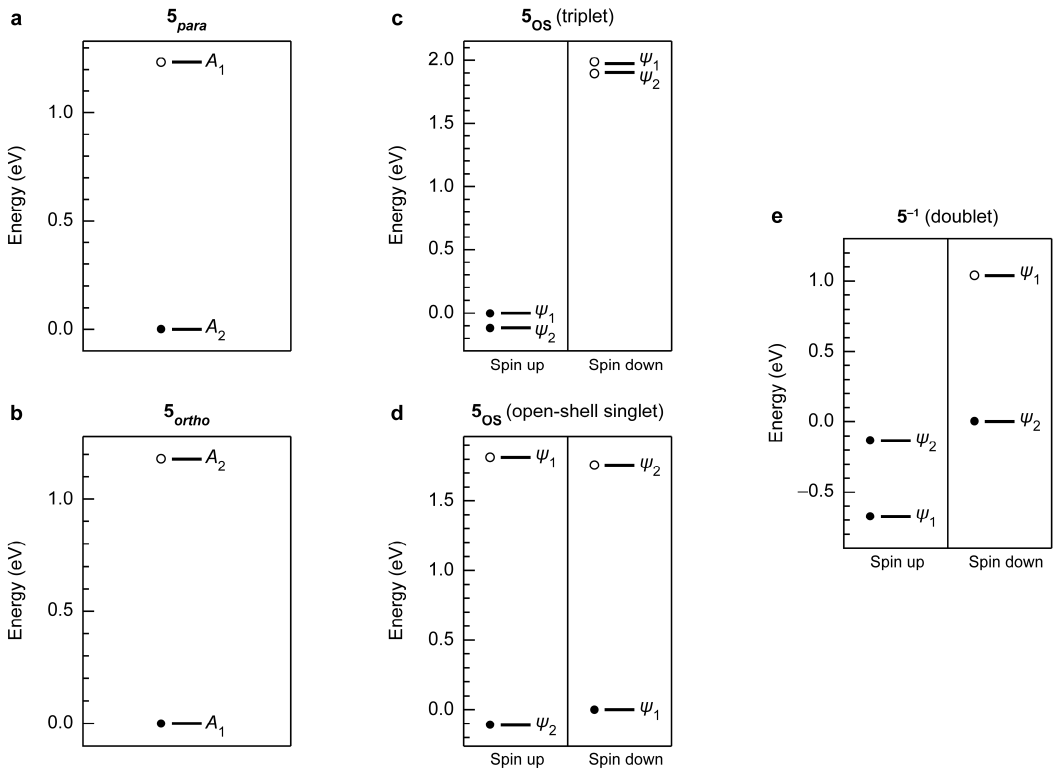

Supplementary Fig. 5 | Density functional theory calculations on indeno[1,2–*a*]fluorene. a,b, Frontier orbital spectrum of $\mathbf{5}_{para}$ (**a**) and $\mathbf{5}_{ortho}$ (**b**). For $\mathbf{5}_{ortho}$, A_1 and A_2 correspond to HOMO and LUMO, respectively, whereas for $\mathbf{5}_{para}$, A_2 is the HOMO and A_1 is the LUMO. Wave functions of A_1 and A_2 are shown in Fig. 3a in the main text. **c,d,** Frontier orbital spectrum of $\mathbf{5}_{OS}$ in the triplet (**c**) and the open-shell singlet (**d**) configurations. ψ_1 and ψ_2 denote SOMOs of the open-shell states. Wave functions of ψ_1 and ψ_2 are shown in Fig. 2a in the main text. For both the triplet and open-shell singlet configurations, ψ_1 is the highest-energy occupied orbital and ψ_2 is the lowest-energy unoccupied orbital. **e,** Frontier orbital spectrum of $\mathbf{5}^{-1}$. In the anionic state, ψ_2 becomes doubly occupied and ψ_1 is the SOMO. Filled and empty circles denote occupied and empty orbitals, respectively. For each panel, zero of the energy axis has been aligned to the respective highest-energy occupied orbital.

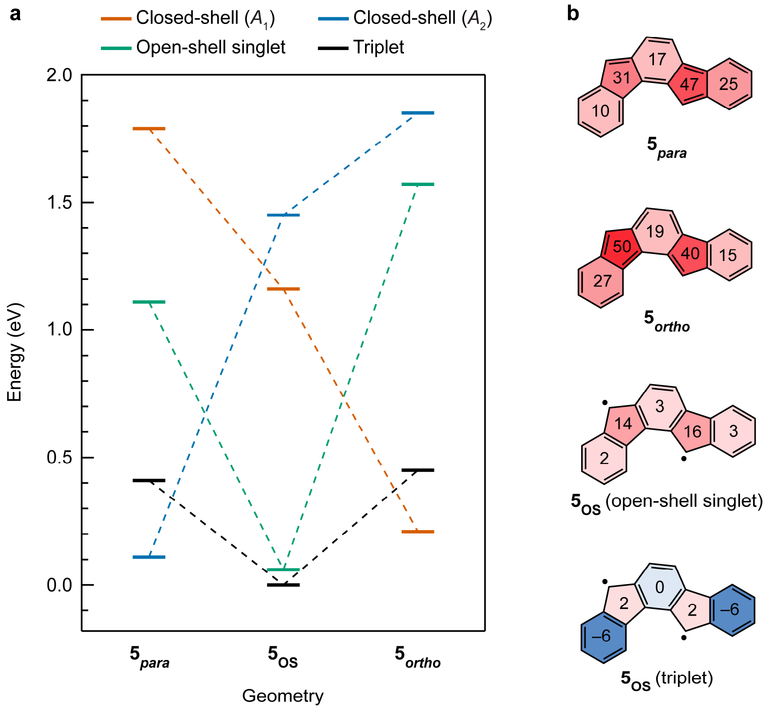

Supplementary Fig. 6 | QD-NEVPT2 and NICS calculations on indeno[1,2-*a*]fluorene. a, QD-NEVPT2 energies of four electronic configurations of $\mathbf{5^0}$, evaluated at three DFT-optimized geometries labeled on the x-axis, namely, $\mathbf{5_{OS}}$, $\mathbf{5}_{\boldsymbol{ortho}}$, and $\mathbf{5}_{\boldsymbol{para}}$. The four electronic configurations are, closed-shell (A_1): closed-shell configuration with A_1 mostly occupied and A_2 mostly unoccupied, closed-shell (A_2): closed-shell configuration with A_2 mostly occupied and A_1 mostly unoccupied, triplet: open-shell triplet configuration, with A_1 and A_2 mostly singly occupied, and open-shell singlet: open-shell singlet configuration, with A_1 and A_2 mostly singly occupied. Zero of the energy axis corresponds to the state with $\mathbf{5_{OS}}$ geometry and triplet configuration. We note that all states have multireference character, and the labeling reflects the determinant with the dominant contribution to the multireference wavefunction (the weight of the main determinant being around 60–70%, with the rest accounting for contribution from the other states). DFT geometry optimization of **5** in the triplet and open-shell singlet configurations yields very similar geometries, with the calculated bond lengths of both geometries differing by less than 0.01 Å. Single-point multireference calculations find that in the DFT-optimized triplet geometry (that is, $\mathbf{5_{OS}}$), both the triplet and open-shell singlet configurations have the lowest energies (the latter is 10 meV below the energy of the open-shell singlet configuration evaluated at the DFT-optimized unrestricted singlet geometry). Therefore, we consider $\mathbf{5_{OS}}$ as the optimal geometry for both the triplet and open-shell singlet configurations. Given the small energy differences between the triplet and singlet electronic configurations of **5**, low-bias spectroscopic signatures such as spin excitations may be experimentally observed. However, the high mobility of **5** on bilayer NaCl precludes the employment of larger tunneling currents that are typically required to detect inelastic processes such as spin excitations. Note that while we cannot distinguish between the triplet and open-shell singlet electronic configurations of **5** in our experiments, future experiments employing electron spin resonance[6] or alternate-charging[7,8] STM may be able to distinguish between the two configurations. From our (single-reference) DFT calculations, $\mathbf{5}_{\boldsymbol{para}}$ and $\mathbf{5}_{\boldsymbol{ortho}}$ are 0.40 and 0.43 eV, respectively, higher than $\mathbf{5_{OS}}$, while from multireference QD-NEVPT2 calculations, $\mathbf{5}_{\boldsymbol{para}}$ and $\mathbf{5}_{\boldsymbol{ortho}}$ are 0.11 and 0.21 eV, respectively, higher than $\mathbf{5_{OS}}$. Thus, compared to DFT calculations, the relative energies of $\mathbf{5}_{\boldsymbol{para}}$ and $\mathbf{5}_{\boldsymbol{ortho}}$ are lowered by 0.29 and 0.22 eV, respectively, in the QD-NEVPT2 calculations. **b,** Results of NICS calculations on **5**. $NICS(0)_{iso}$ values of **5** in different states are calculated at the B3LYP/def2-TZVP level of theory. Negative (positive) $NICS(0)_{iso}$ values are indicative of aromaticity (antiaromaticity), while values close to zero suggest nonaromaticity. $\mathbf{5_{OS}}$ in the triplet configuration exhibits local aromaticity at the terminal benzenoid rings, whereas $\mathbf{5_{OS}}$ in the open-shell singlet configuration, $\mathbf{5}_{\boldsymbol{para}}$ and $\mathbf{5}_{\boldsymbol{ortho}}$ all display antiaromaticity[9–11]. Thereby, switching between $\mathbf{5_{OS}}$ (triplet) and $\mathbf{5}_{\boldsymbol{para}}$, as in our experiments, entails a substantial change in the aromaticity of **5**.

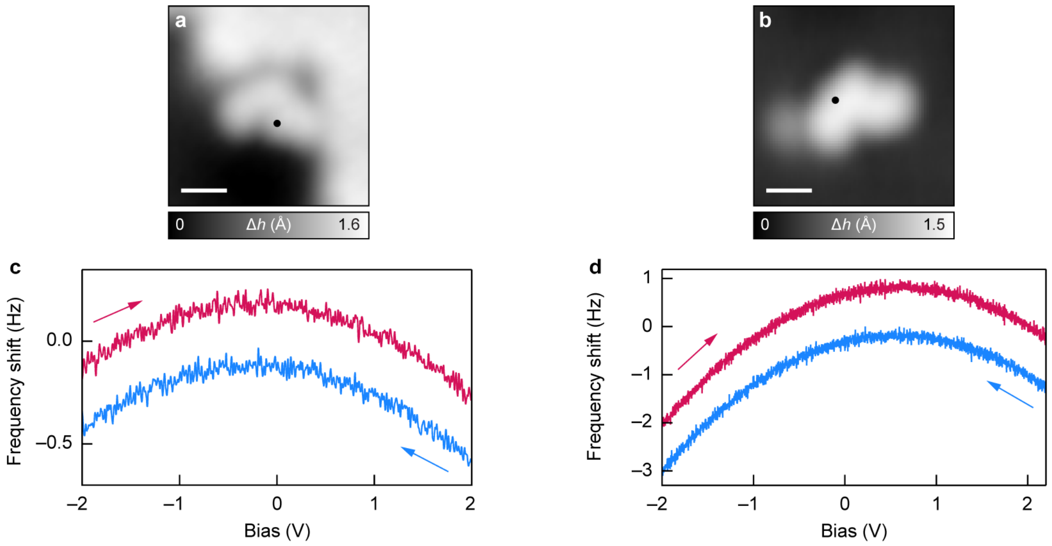

Supplementary Fig. 7 | Kelvin probe force spectroscopy on indeno[1,2-*a*]fluorene on bilayer NaCl/Au(111). a,b, In-gap STM images of $\mathbf{5}_{\mathrm{OS}}$ (**a**) and $\mathbf{5}_{para}$ (**b**) on bilayer NaCl/Au(111). The molecule in **a** is shown in Fig. 2f in the main text, while the molecule in **b** is shown in the STM images of Fig. 3d in the main text. Scanning parameters: V = 0.2 V, I = 0.3 pA (**a**) and 0.5 pA (**b**). Δ*h* denotes the tip height. **c,d,** Constant-height Δ*f*(*V*) spectra acquired on $\mathbf{5}_{\mathrm{OS}}$ (**c**) and $\mathbf{5}_{para}$ (**d**). Open feedback parameters: V = 2 V, I = 0.17 pA (**c**) and V = 2.2 V, I = 0.15 pA (**d**). Colored arrows indicate the bias sweep directions for the corresponding Δ*f*(*V*) curves. Bias sweeps are as follows, forward sweep: 2 V to –2 V (blue) and backward sweep: –2 V to 2 V (red) (**c**); forward sweep: 2.2 V to –2 V (blue) and backward sweep: –2 V to 2.2 V (red) (**d**). The backward Δ*f*(*V*) curve is offset by 0.3 Hz (**c**) and 1 Hz (**d**) relative to the corresponding forward curve for visual clarity. Acquisition positions of the Δ*f*(*V*) spectra are marked by a filled circle in the corresponding in-gap STM images. The acquisition positions shown in **a** and **b** also correspond to the acquisition positions for the *I*(*V*) curves in Fig. 2e and Fig. 3c in the main text, respectively. Abrupt steps in Δ*f*(*V*) spectra of atoms[12] and molecules[13] on insulating surfaces have been shown to arise from charge-state transitions. The lack of any steps in the Δ*f*(*V*) spectra in the present case, in conjunction with the absence of NaCl/Au(111) interface-state scattering by **5** (not shown), signifies the neutral charge state of **5** on bilayer NaCl/Au(111) for both $\mathbf{5}_{\mathrm{OS}}$ and $\mathbf{5}_{para}$. Scale bars: 5 Å.

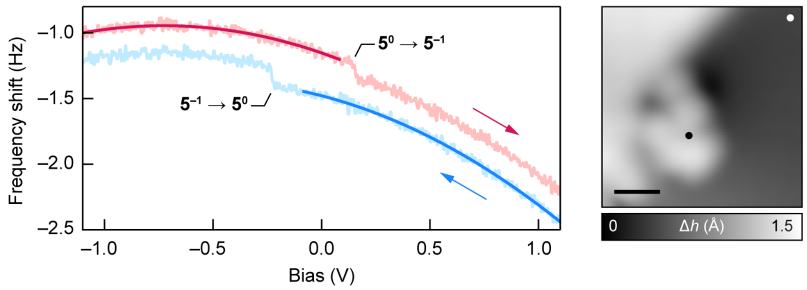

Δz (Å)	LCPD above 5^{-1} (V)	LCPD above 5^{0} (V)	LCPD above bilayer NaCl (V)
0	–0.529	–0.727	–0.866
–0.5	–0.538	–0.746	–0.852
–1.0	–0.520	–0.726	–0.846

Supplementary Fig. 8 | Kelvin probe force spectroscopy on indeno[1,2-*a*]fluorene on bilayer NaCl/Ag(111). Constant-height Δ*f*(*V*) spectra acquired on **5** on bilayer NaCl/Ag(111). Open feedback parameters: V = 1.1 V, I = 0.2 pA. Forward sweep: 1.1 V to –1.1 V (blue) and backward sweep: –1.1 V to 1.1 V (red). The backward Δ*f*(*V*) curve is offset by 0.2 Hz relative to the forward curve. Also shown are parabolic fits to the Δ*f*(*V*) curves on **5** in the two states. The local contact potential difference (LCPD) is extracted from the abscissas of the vertices of the Δ*f*(*V*) parabolas for three Δ*z* values. A larger (more

positive) LCPD indicates a more negatively charged species[14]. As shown in the table, for the entire Δz range, LCPD values above **5** for the state corresponding to $V > 0.2$ V (**5**$^{-1}$) are larger than for the state corresponding to $V < -0.2$ V (**5**^{0}). This indicates that the steps in the Δ$f(V)$ spectra correspond to charge state transitions. The identity of the states as **5**$^{-1}$ and **5**^{0} is confirmed by the observation of the presence (**5**$^{-1}$) or absence (**5**^{0}) of NaCl/metal interface-state scattering by the respective species (Supplementary Fig. 9). LCPD values on bilayer NaCl are shown for reference. The hysteretic behavior of the charging (**5**^{0} → **5**$^{-1}$)/discharging (**5**$^{-1}$ → **5**^{0}) process relates to the reorganization energy[13]. Within the hysteresis loop, **5** is charge bistable and can be imaged both in the neutral and anionic states. Open feedback parameters: V = 1.1 V, I = 0.2 pA. Acquisition positions of the Δ$f(V)$ spectra on **5** and on bilayer NaCl (not shown here) are marked by filled circles in the in-gap STM image of **5**$^{-1}$ (V = 0.2 V, I = 1 pA). Note that in the neutral state, this species corresponds to **5**$_{OS}$. Scale bar: 5 Å. On bilayer NaCl/Ag(111) and bilayer NaCl/Cu(111) (Supplementary Fig. 9), **5** exhibits charge bistability. This contrasts with **2**, an isomer of **5**, which was found to adopt a neutral charge state on bilayer NaCl/Cu(111)[3]. This difference results from the different frontier orbital gaps of **2** and **5**, and the relative alignment of the frontier orbitals with respect to the Fermi level of the surface[15].

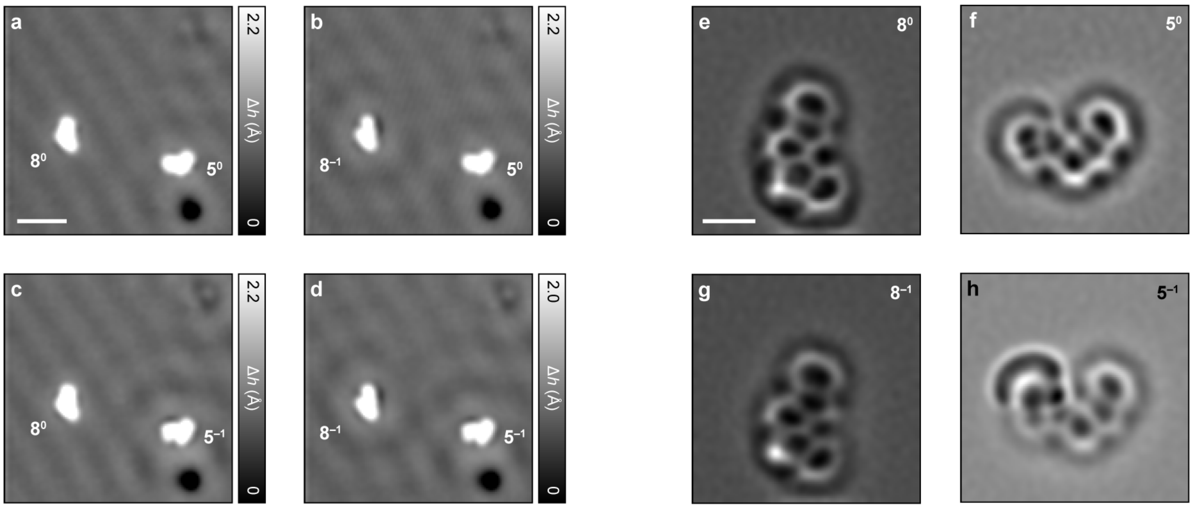

Supplementary Fig. 9 | Observation of NaCl/Cu(111) interface-state scattering by charged species. a–d, STM images of **5** and monoradical **8** on bilayer NaCl/Cu(111) in their neutral and negative states: **5**^{0} and **8**^{0} (**a**), **5**^{0} and **8**$^{-1}$ (**b**), **5**$^{-1}$ and **8**^{0} (**c**), and **5**$^{-1}$ and **8**$^{-1}$ (**d**). Scattering of NaCl/Cu(111) interface state[15,16] is observed by the charged species **5**$^{-1}$ and **8**$^{-1}$. Scanning parameters: V = 0.1 V, I = 0.3 pA. The bias voltage (V = 0.1 V) is chosen to be above the onset[16] of NaCl/Cu(111) interface state at approximately –0.2 V. **e–h,** Laplace-filtered AFM images of **5** (**f,h**) and **8** (**e,g**) in their neutral and negative states. STM set point: V = 0.2 V, I = 0.5 pA on bilayer NaCl, Δz = –0.4 Å. In the absence of any adjacent third layer NaCl island, adsorbates or defects on the surface, **5** moves under the influence of the tip during image acquisition[17]. This causes the apparent bisection of the leftmost ring in the AFM images of both **5**^{0} and **5**$^{-1}$. On the defect-free NaCl surface, **5** always showed this movement and in addition, exhibited mobility when increasing the bias voltage to obtain orbital density images. For these reasons, we could not characterize the electronic configuration of **5** on the defect-free NaCl surface. In general, **5** seems to be less stably adsorbed than its isomer **2** (ref. [3]) on NaCl. This may be related to the lower symmetry of **5**, and the symmetry and geometry of **5** not matching well with the NaCl surface[17]. It could also be related to the existence of different metastable adsorption sites and orientations of **5** on NaCl, possibly allowing smaller movement steps for translations and rotations. Scale bars: 20 Å (**a–d**) and 5 Å (**e–h**).

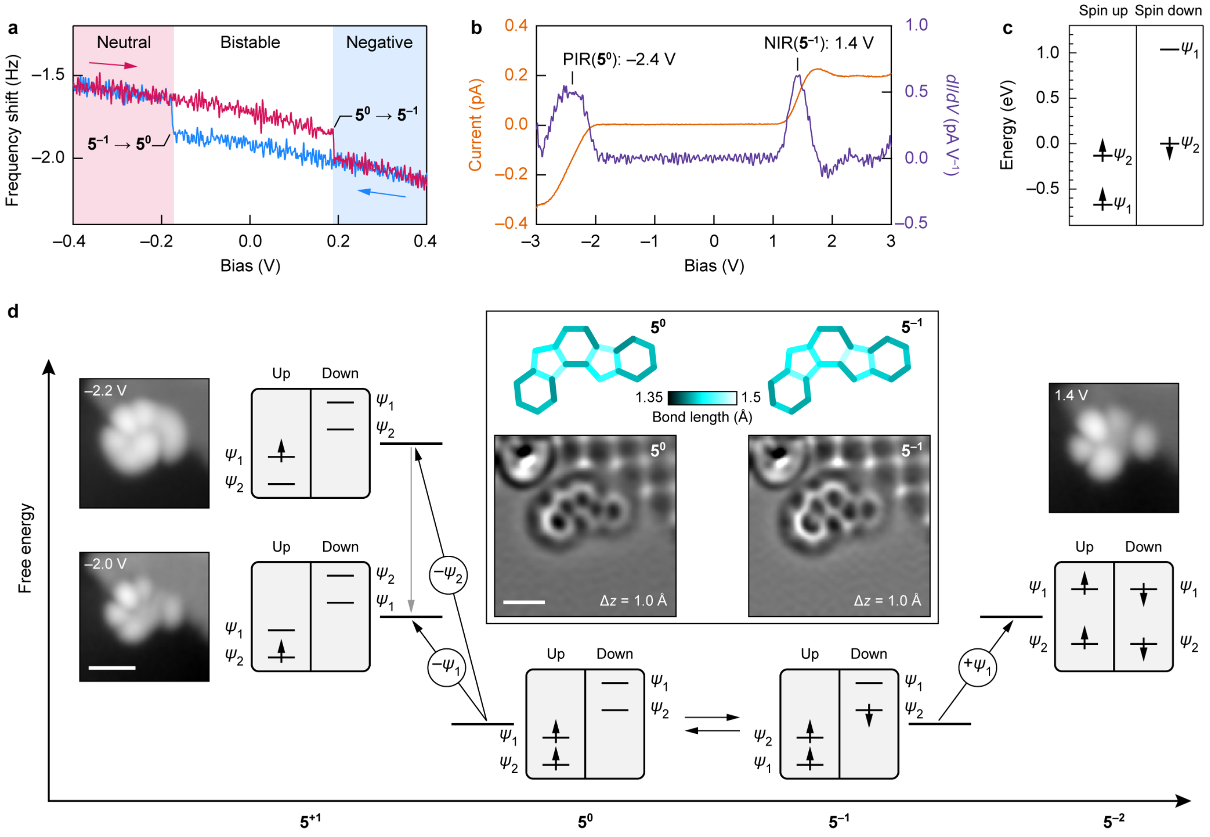

Supplementary Fig. 10 | Characterization of open-shell indeno[1,2-*a*]fluorene on bilayer NaCl/Ag(111). a, Constant-height Δ*f*(*V*) spectra acquired on **5** on bilayer NaCl/Ag(111). Open feedback parameters: *V* = 0.4 V, *I* = 0.2 pA. Charge transitions between $\mathbf{5^0}$ and $\mathbf{5^{-1}}$ are indicated. Forward sweep: 0.4 V to –0.4 V (blue) and backward sweep: –0.4 V to 0.4 V (red). **b,** Constant-height *I*(*V*) spectrum acquired on **5**, along with the corresponding *dI/dV*(*V*) spectrum. Open feedback parameters: *V* = 3 V, *I* = 0.15 pA. Given that **5** is in a neutral state for $V < -0.17$ V and in an anionic state for $V > 0.19$ V, the peaks at –2.4 V and 1.4 V correspond to the PIR of $\mathbf{5^0}$ and NIR of $\mathbf{5^{-1}}$, respectively. In the many-body picture shown in **d**, the NIR corresponds to transitions between $\mathbf{5^{-1}}$ and the dianionic ($\mathbf{5^{-2}}$) state of **5**. **c,** DFT-calculated frontier orbital spectrum of $\mathbf{5^{-1}}$. Zero of the energy axis has been aligned to the highest-energy occupied orbital, namely, $\psi_{2\downarrow}$. **d,** Scheme of many-body transitions associated to the measured ionic resonances, along with STM images of **5** at biases where the corresponding transitions become accessible. STM images at –2 V and –2.2 V show the orbital density of ψ_1, and superposition of ψ_1 and ψ_2, respectively; and the STM image at 1.4 V shows orbital density of ψ_1. Scanning parameters: *I* = 0.15 pA (*V* = –2 V and –2.2 V) and 0.2 pA (*V* = 1.4 V). Inset, DFT-calculated bond lengths of $\mathbf{5^0}$ (corresponding to $\mathbf{5_{OS}}$) and $\mathbf{5^{-1}}$ (top), and Laplace-filtered AFM images of $\mathbf{5^0}$ and $\mathbf{5^{-1}}$ (bottom). STM set point: *V* = 0.2 V, *I* = 0.5 pA on third layer NaCl island. Note that in the neutral state, the species corresponds to $\mathbf{5_{OS}}$. Scale bars: 5 Å (AFM images) and 10 Å (STM images).

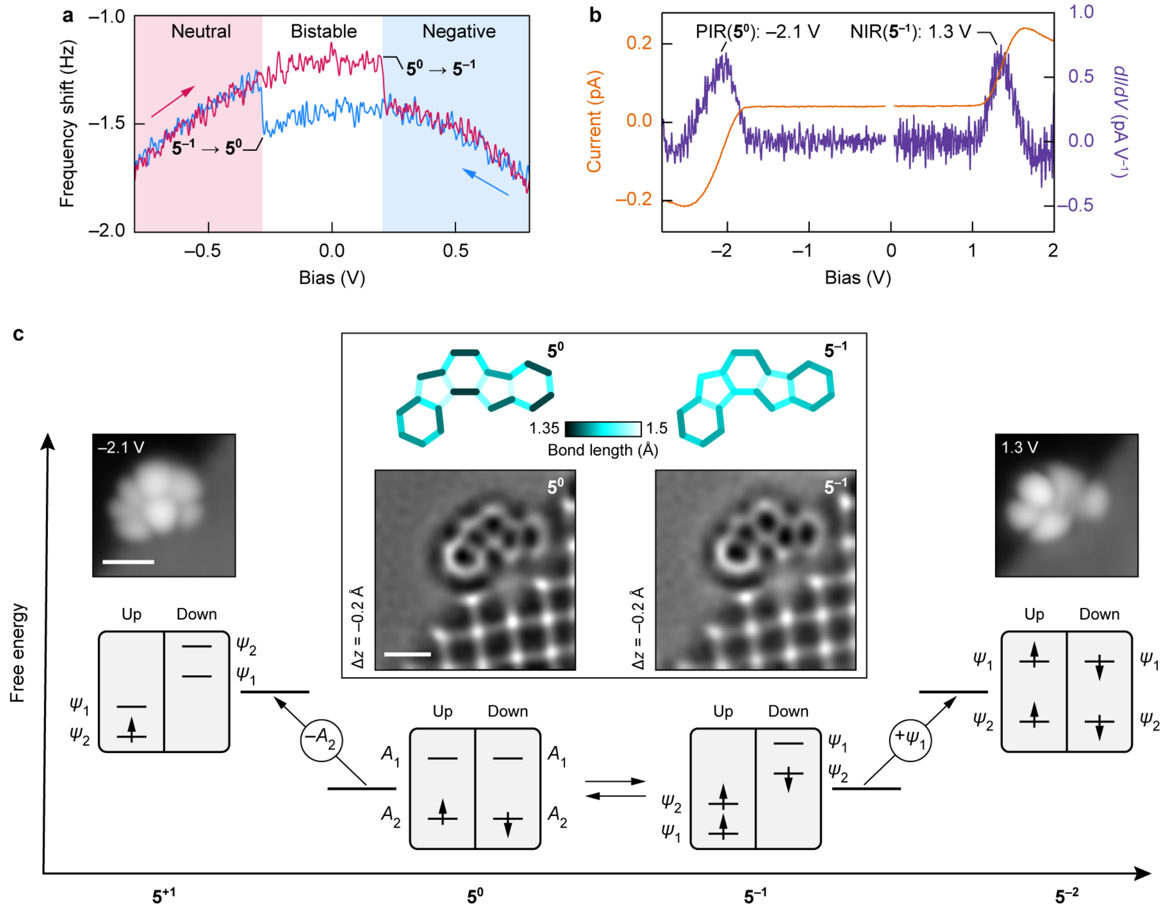

Supplementary Fig. 11 | Characterization of closed-shell indeno[1,2-*a*]fluorene on bilayer NaCl/Cu(111). a, Constant-height $\Delta f(V)$ spectra acquired on **5** on bilayer NaCl/Cu(111). Open feedback parameters: $V = 0.8$ V, $I = 0.2$ pA. Charge transitions between $\mathbf{5}^0$ and $\mathbf{5}^{-1}$ are indicated. Forward sweep: 0.8 V to –0.8 V (blue) and backward sweep: –0.8 V to 0.8 V (red). **b,** Constant-height $I(V)$ spectra acquired on **5**, along with the corresponding $dI/dV(V)$ spectra. Open feedback parameters: $V = -2.8$ V, $I = 0.2$ pA (negative bias side) and $V = 2$ V, $I = 0.2$ pA (positive bias side). **c,** Scheme of many-body transitions associated to the measured ionic resonances, along with STM images of **5** at biases where the corresponding transitions become accessible. STM images at –2.1 V and 1.3 V show orbital densities of A_2 and ψ_1, respectively. Scanning parameters: $I = 0.25$ pA ($V = -2.1$ V) and 0.3 pA ($V = 1.3$ V). Inset, DFT-calculated bond lengths of $\mathbf{5}^0$ (corresponding to $\mathbf{5}_{para}$) and $\mathbf{5}^{-1}$ (top), and Laplace-filtered AFM images of $\mathbf{5}^0$ and $\mathbf{5}^{-1}$ (bottom). STM set point: $V = 0.2$ V, $I = 0.5$ pA on bilayer NaCl. Note that in the neutral state, the species corresponds to $\mathbf{5}_{para}$. Scale bars: 5 Å (AFM images) and 10 Å (STM images).

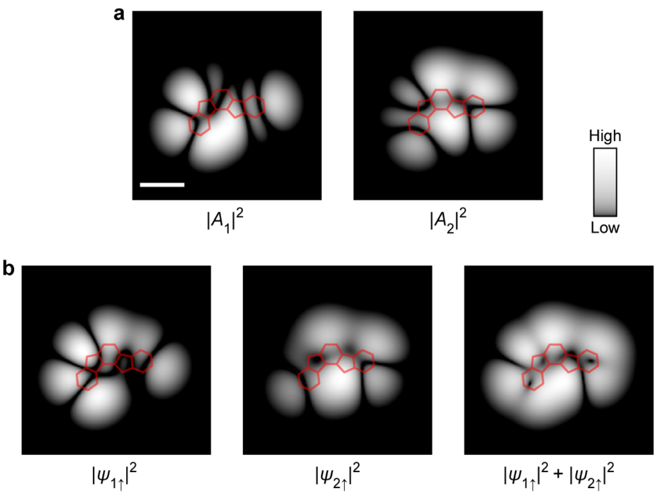

Supplementary Fig. 12 | Calculated local density of states maps of 5. a, Constant-height tight-binding local density of states maps of A_1 and A_2. **b,** Constant-height mean-field Hubbard local density of states maps of $\psi_{1\uparrow}$, $\psi_{2\uparrow}$, and superposition of the densities of $\psi_{1\uparrow}$ and $\psi_{2\uparrow}$. All maps are calculated at a height of 7 Å above the molecular plane and are shown in a logarithmic color scale, which (due to the exponential dependence of the tunneling current on the distance) facilitates comparison with constant-current STM images, shown in Figs. 2 and 3. Scale bar: 5 Å.

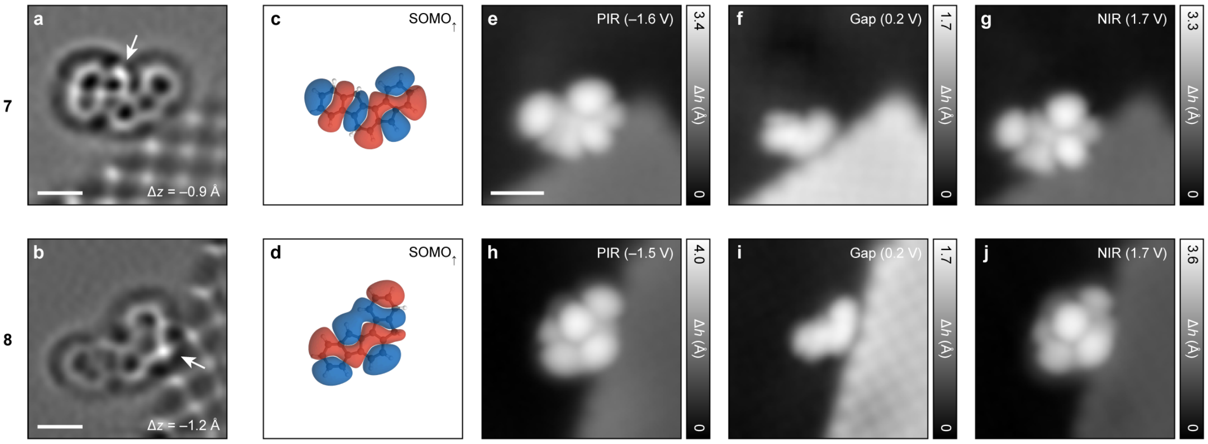

Supplementary Fig. 13 | Characterization of monoradical species on bilayer NaCl/Au(111). a,b, Laplace-filtered AFM images of the monoradical species **7** (**a**) and **8** (**b**). Doubly hydrogenated pentagon apexes are indicated with arrows. In these measurements, the monoradical species exhibit movement under the influence of the tip during image acquisition. STM set point: $V = 0.2$ V, $I = 0.5$ pA on bilayer NaCl. **c,d,** DFT-calculated wave functions of the SOMO of **7** (**c**) and **8** (**d**) (isovalue: 0.002 e^{-} Å^{-3}). Only the spin up (occupied) level of the SOMOs is shown. DFT calculations predict a doublet ground state of **7** and **8** in the neutral charge state. **e–g,** STM images of **7** acquired at the PIR (**e**), in gap (**f**) and at the NIR (**g**). **h–j,** STM images of **8** acquired at the PIR (**h**), in gap (**i**) and at the NIR (**j**). Scanning parameters: I = 0.15 pA (**e,g**), 0.2 pA (**h,j**) and 0.3 pA (**f,i**). Note that in **f** and **g**, the molecule had translated along the edge of the third layer NaCl island toward the bottom-left of the scan frame. Scale bars: 5 Å (**a,b**) and 10 Å (**e–j**). Gas-phase DFT calculations predict **7** to be roughly 50 meV lower in energy than **8**. Among 17 monoradical species that were generated and analyzed on the metal and NaCl surfaces, eight corresponded to **7** and nine corresponded to **8**, indicating that they are formed with roughly equal probabilities from voltage pulse-induced dehydrogenation of **6**.

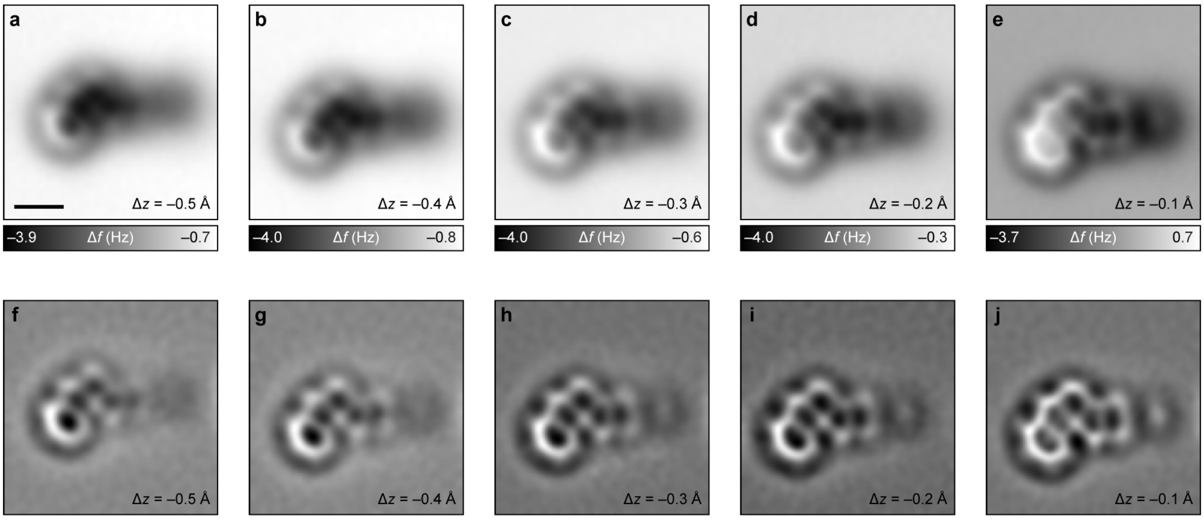

Supplementary Fig. 14 | Height-dependent AFM imaging of closed-shell indeno[1,2-*a*]fluorene on bilayer NaCl/Cu(111). a–e, AFM images of $\mathbf{5}_{para}$ at different tip heights. The tip is 0.4 Å closer to **5** in **e** than in **a**. **f–j,** Corresponding Laplace-filtered AFM images. STM set point: V = 0.2 V, I = 0.5 pA on bilayer NaCl. Scale bar: 5 Å.

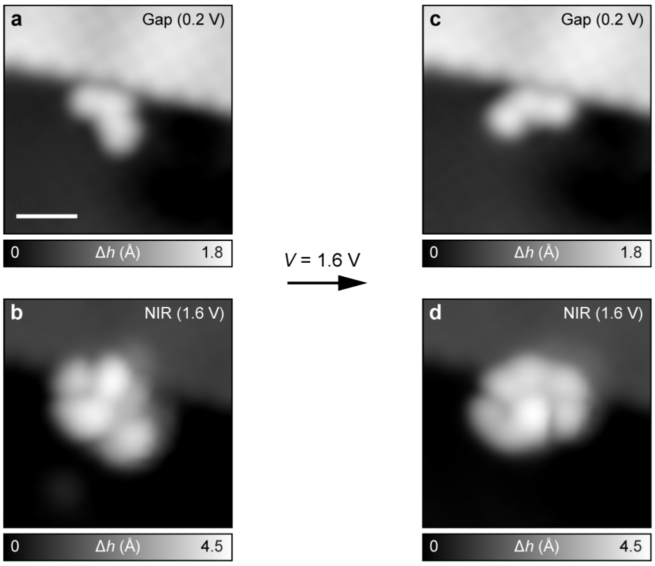

Supplementary Fig. 15 | Switching between open- and closed-shell states of indeno[1,2-*a*]fluorene on bilayer NaCl/Au(111). a,b, STM images of $\mathbf{5}_{para}$ acquired in gap (**a**) and at the NIR (**b**). STM image at the NIR shows orbital density of A_1, evidencing that the species corresponds to $\mathbf{5}_{para}$. Subsequent scanning at V = 1.6 V led to a change in adsorption site, as observed in the in-gap STM image in **c**. **d,** STM imaging at the NIR now shows superposition of ψ_1 and ψ_2, evidencing switching to $\mathbf{5}_{\mathrm{OS}}$. Scanning parameters: I = 0.5 pA (**a**), 0.15 pA (**b**), 0.4 pA (**c**) and 0.2 pA (**d**). Scale bar: 10 Å.

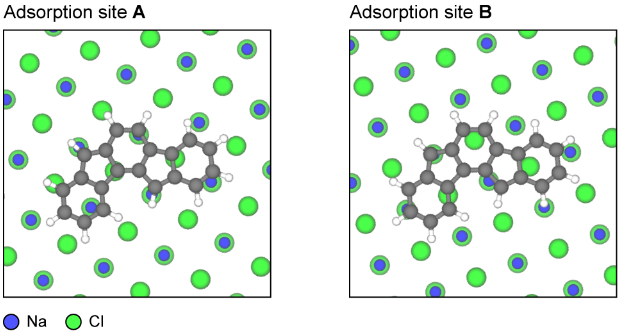

Adsorption site	Energy, $\mathbf{5}_{OS}$ (eV)	Energy, $\mathbf{5}_{para}$ (eV)	Energy, $\mathbf{5}_{ortho}$ (eV)
**A**	0	0.24	0.37
**B**	0.03	0.38	0.32

Supplementary Fig. 16 | On-surface DFT calculations. DFT-optimized adsorption sites of **5** on NaCl (labeled **A** and **B**). The relative energies of $\mathbf{5}_{OS}$, $\mathbf{5}_{para}$ and $\mathbf{5}_{ortho}$ states in sites **A** and **B** are tabulated. The two adsorption sites on bilayer NaCl and the relative stability of the corresponding states in the table were also confirmed with planewave DFT using periodic boundary conditions with one molecule adsorbed on a 5×5 surface slab.

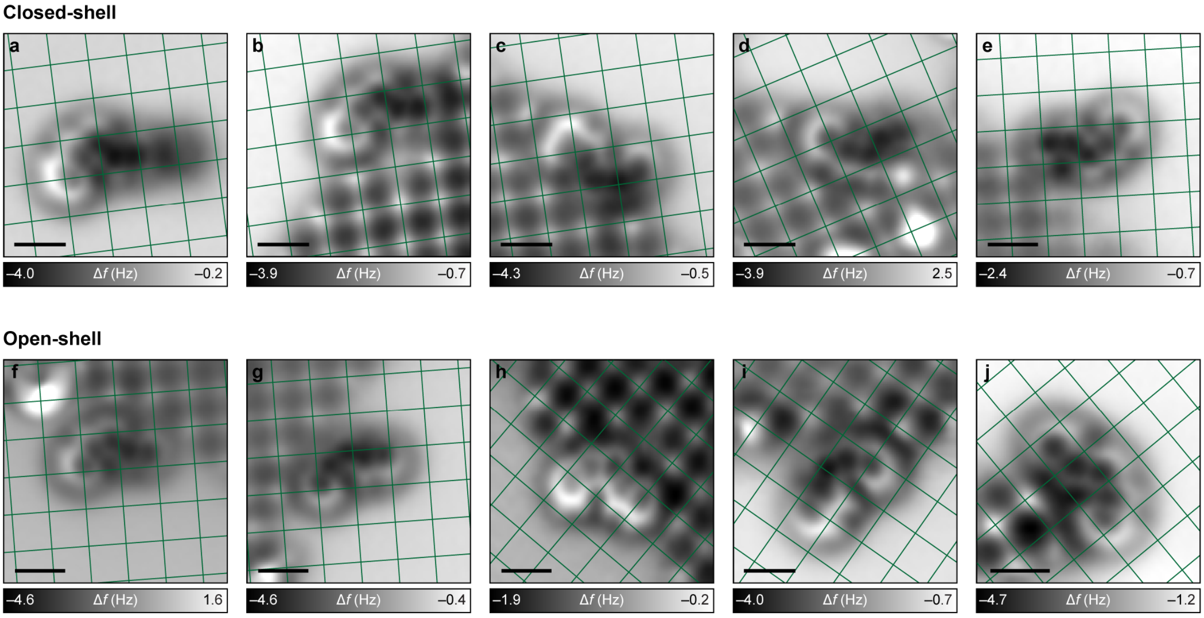

Supplementary Fig. 17 | Experimental adsorption site determination of 5 using AFM data. a–e, AFM images of five $\mathbf{5}_{para}$ species. **f–j,** AFM images of five $\mathbf{5}_{OS}$ species. The overlaid lattices (in green) visualize the NaCl lattice. Crossing points correspond to Na^{+} (Cl^{-}) sites of the second (third) NaCl layer. **5** is adsorbed on bilayer NaCl/Cu(111) in **a**–**d**, **i** and **j**, on bilayer NaCl/Au(111) in **e** and **h**, and on bilayer NaCl/Ag(111) in **f** and **g**. Apart from **a**, where **5** is adsorbed next to a defect on the NaCl surface, **5** is adsorbed next to a third layer NaCl island in all cases. Scale bars: 5 Å.

Supplementary Note 1. Stabilization of and switching between open- and closed-shell states of 5.

The following experimental observations are made with regards to switching between $\mathbf{5}_{\mathrm{OS}}$ and $\mathbf{5}_{para}$:

1. Switching only takes place when **5** is moved on NaCl, thereby changing its adsorption site. However, in some cases, there is no switching despite movement of **5**.
2. On bilayer NaCl/Ag(111) and Cu(111), where we can reversibly switch the charge state of **5** multiple times between $\mathbf{5}^{0}$ and $\mathbf{5}^{-1}$ (with the $\mathbf{5}^{-1}$ charge state being equivalent, independently of coming from the $\mathbf{5}_{\mathrm{OS}}$ or $\mathbf{5}_{para}$ neutral state), we invariably observe the same neutral ground state ($\mathbf{5}_{\mathrm{OS}}$ or $\mathbf{5}_{para}$) when returning to $\mathbf{5}^{0}$, provided there was no movement of the molecule.

These observations show that by changing the adsorption site of **5** on NaCl, its ground state can be changed, but without changing the adsorption site the ground state of **5** does not change, even if returning from the anionic ($\mathbf{5}^{-1}$) state that is equivalent for both the (neutral) ground states ($\mathbf{5}_{\mathrm{OS}}$ or $\mathbf{5}_{para}$). This leads us to the assumption that stabilization of the open- or closed-shell state of **5** depends on its adsorption site on the NaCl surface. To capture this theoretically, we performed DFT calculations of **5** on a defect-free NaCl(100) surface. Supplementary Fig. 16 shows the DFT-optimized adsorption sites of **5** on NaCl, labeled as **A** and **B**. Sites **A** and **B** are stable and metastable, respectively, for $\mathbf{5}_{\mathrm{OS}}$ and $\mathbf{5}_{para}$. The relative energies of the three states of **5** in site **A** are: $\mathbf{5}_{\mathrm{OS}}$ (0 eV) < $\mathbf{5}_{para}$ (0.24 eV) < $\mathbf{5}_{ortho}$ (0.37 eV); while the energies of the three states in site **B**, relative to the $\mathbf{5}_{\mathrm{OS}}$ state in site **A**, are: $\mathbf{5}_{\mathrm{OS}}$ (0.03 eV) < $\mathbf{5}_{ortho}$ (0.32 eV) < $\mathbf{5}_{para}$ (0.38 eV).

Supplementary Fig. 17 shows the experimentally-determined adsorption sites of five $\mathbf{5}_{para}$ species (Supplementary Fig. 17a–e) and five $\mathbf{5}_{\mathrm{OS}}$ species (Supplementary Fig. 17f–j). For $\mathbf{5}_{para}$, all adsorption sites correspond to site **A**, except the one in Supplementary Fig. 17c that does not correspond to sites **A** or **B** (out of total eight $\mathbf{5}_{para}$ species analyzed, seven were found to adsorb in site **A**). For $\mathbf{5}_{\mathrm{OS}}$, while the adsorption site in Supplementary Fig. 17f corresponds to site **A**, the rest of the adsorption sites do not correspond to sites **A** or **B** (out of total seven $\mathbf{5}_{\mathrm{OS}}$ species analyzed, only one was found to adsorb in site **A**, with the rest not adsorbing in sites **A** or **B**). This poses a conundrum: in site **A**, DFT predicts $\mathbf{5}_{\mathrm{OS}}$ to be the ground state, with $\mathbf{5}_{para}$ 0.24 eV higher in energy; while experimentally, nearly all species found in site **A** correspond to $\mathbf{5}_{para}$, with only one $\mathbf{5}_{\mathrm{OS}}$ species found in site **A**. The resolution to this conundrum may come from more accurate multireference calculations, which, compared to single-reference DFT calculations in gas phase, substantially lower the energies of the closed-shell states relative to the open-shell state (see Supplementary Fig. 6). While multireference calculations are unfeasible on surfaces, we may assume that as in gas phase, on-surface DFT calculations will overestimate the energies of the closed-shell states relative to the open-shell state. Qualitatively, we propose that the DFT-calculated energies of $\mathbf{5}_{para}$ and $\mathbf{5}_{ortho}$ on NaCl are lowered to the extent that $\mathbf{5}_{para}$ would become the ground state in site **A**, that is, by more than 0.24 eV. Note that in site **A**, $\mathbf{5}_{para}$ is found 0.24 eV above $\mathbf{5}_{\mathrm{OS}}$, but in site **B**, $\mathbf{5}_{para}$ is found 0.35 eV (and $\mathbf{5}_{ortho}$ 0.29 eV) above $\mathbf{5}_{\mathrm{OS}}$. Therefore, in site **B**, $\mathbf{5}_{\mathrm{OS}}$ would remain as the ground state if the energies of the closed-shell states are not lowered by more than 0.29 eV. Such magnitude of corrections to single-reference DFT energies seem reasonable, given that the gas-phase energies of the closed-shell states are lowered by 0.22 eV ($\mathbf{5}_{ortho}$) and 0.29 eV ($\mathbf{5}_{para}$) relative to the open-shell state, when using a multireference approach (Supplementary Fig. 6).

These arguments would account for our experimental observation that nearly all $\mathbf{5}_{para}$ species are found in site **A**, while in site **B**, $\mathbf{5}_{\mathrm{OS}}$ would remain as the ground state. Also note that up until now, our discussion from a theoretical standpoint has been focused on **5** on a defect-free NaCl surface. Experimentally, **5** is stably adsorbed only when it is adjacent to a third layer NaCl island, an adsorbate, or a defect on the NaCl surface, each of which will influence the adsorption energetics of **5**. Therefore, deviations from adsorption site **A** for $\mathbf{5}_{para}$ species (as in Supplementary Fig. 17c) can likely be accounted for by the influence of adjacent third layer NaCl islands, adsorbates, or defects on the surface. We find a larger variation in the experimentally-determined adsorption sites of $\mathbf{5}_{\mathrm{OS}}$ (Supplementary Fig. 17f–j) compared to $\mathbf{5}_{para}$, which points toward a shallower adsorption energy landscape of $\mathbf{5}_{\mathrm{OS}}$ on NaCl.

The important finding and observation of on-surface DFT calculations is that the energy differences between **5**$_{OS}$ and **5**$_{para}$ change substantially at different adsorption sites. Likely, these changes are even more pronounced for adsorption near third layer NaCl islands, adsorbates, or defects, compared to the defect-free NaCl surface.

3. References

1. Hsiao, C.-C., Lin, Y.-K., Liu, C.-J., Wu, T.-C. & Wu, Y.-T. Synthesis of Methylene-Bridge Polyarenes through Palladium-Catalyzed Activation of Benzylic Carbon-Hydrogen Bond. *Adv. Synth. Catal.* **352**, 3267–3274 (2010).
2. Gross, L., Mohn, F., Moll, N., Liljeroth, P. & Meyer, G. The Chemical Structure of a Molecule Resolved by Atomic Force Microscopy. *Science* **325**, 1110–1114 (2009).
3. Majzik, Z. *et al.* Studying an antiaromatic polycyclic hydrocarbon adsorbed on different surfaces. *Nat. Commun.* **9**, 1198 (2018).
4. Romaner, L., Nabok, D., Puschnig, P., Zojer, E. & Ambrosch-Draxl, C. Theoretical study of PTCDA adsorbed on the coinage metal surfaces, Ag(111), Au(111) and Cu(111). *New J. Phys.* **11**, 053010 (2009).
5. Di Giovannantonio, M. *et al.* On-Surface Synthesis of Antiaromatic and Open-Shell Indeno[2,1-*b*]fluorene Polymers and Their Lateral Fusion into Porous Ribbons. *J. Am. Chem. Soc.* **141**, 12346–12354 (2019).
6. Baumann, S. *et al.* Electron paramagnetic resonance of individual atoms on a surface. *Science* **350**, 417–420 (2015).
7. Patera, L. L., Queck, F., Scheuerer, P. & Repp, J. Mapping orbital changes upon electron transfer with tunnelling microscopy on insulators. *Nature* **566**, 245–248 (2019).
8. Fatayer, S. *et al.* Probing Molecular Excited States by Atomic Force Microscopy. *Phys. Rev. Lett.* **126**, 176801 (2021).
9. Dressler, J. J. *et al.* Synthesis of the Unknown Indeno[1,2-a]fluorene Regioisomer: Crystallographic Characterization of Its Dianion. *Angew. Chem. Int. Ed.* **56**, 15363–15367 (2017).
10. Ottosson, H. Exciting excited-state aromaticity. *Nat. Chem.* **4**, 969–971 (2012).
11. Solà, M. Aromaticity rules. *Nat. Chem.* **14**, 585–590 (2022).
12. Steurer, W. *et al.* Manipulation of the Charge State of Single Au Atoms on Insulating Multilayer Films. *Phys. Rev. Lett.* **114**, 036801 (2015).
13. Fatayer, S. *et al.* Reorganization energy upon charging a single molecule on an insulator measured by atomic force microscopy. *Nat. Nanotechnol.* **13**, 376–380 (2018).
14. Gross, L. *et al.* Measuring the Charge State of an Adatom with Noncontact Atomic Force Microscopy. *Science* **324**, 1428–1431 (2009).
15. Swart, I., Sonnleitner, T. & Repp, J. Charge State Control of Molecules Reveals Modification of the Tunneling Barrier with Intramolecular Contrast. *Nano Lett.* **11**, 1580–1584 (2011).
16. Repp, J., Meyer, G. & Rieder, K.-H. Snell's Law for Surface Electrons: Refraction of an Electron Gas Imaged in Real Space. *Phys. Rev. Lett.* **92**, 036803 (2004).
17. Pavliček, N. *et al.* Synthesis and characterization of triangulene. *Nat. Nanotechnol.* **12**, 308–311 (2017).
